# Genome-wide census of ATF4 binding sites and functional profiling of trait-associated genetic variants overlapping ATF4 binding motifs

**DOI:** 10.1371/journal.pgen.1011014

**Published:** 2023-10-31

**Authors:** Tiit Örd, Daima Örd, Priit Adler, Tõnis Örd

**Affiliations:** 1 Institute of Genomics, University of Tartu, Tartu, Estonia; 2 A. I. Virtanen Institute for Molecular Sciences, University of Eastern Finland, Kuopio, Finland; 3 Institute of Computer Science, University of Tartu, Tartu, Estonia; HudsonAlpha Institute for Biotechnology, UNITED STATES

## Abstract

Activating Transcription Factor 4 (ATF4) is an important regulator of gene expression in stress responses and developmental processes in many cell types. Here, we catalogued ATF4 binding sites in the human genome and identified overlaps with trait-associated genetic variants. We probed these genetic variants for allelic regulatory activity using a massively parallel reporter assay (MPRA) in HepG2 hepatoma cells exposed to tunicamycin to induce endoplasmic reticulum stress and ATF4 upregulation. The results revealed that in the majority of cases, the MPRA allelic activity of these SNPs was in agreement with the nucleotide preference seen in the ATF4 binding motif from ChIP-Seq. Luciferase and electrophoretic mobility shift assays in additional cellular models further confirmed ATF4-dependent regulatory effects for the SNPs rs532446 (*GADD45A* intronic; linked to hematological parameters), rs7011846 (*LPL* upstream; myocardial infarction), rs2718215 (diastolic blood pressure), rs281758 (psychiatric disorders) and rs6491544 (educational attainment). CRISPR-Cas9 disruption and/or deletion of the regulatory elements harboring rs532446 and rs7011846 led to the downregulation of *GADD45A* and *LPL*, respectively. Thus, these SNPs could represent examples of GWAS genetic variants that affect gene expression by altering ATF4-mediated transcriptional activation.

## Author summary

Genome-wide association studies (GWAS) have gathered a large amount of data connecting genetic variations (mostly single-nucleotide polymorphisms (SNPs)) to phenotypic traits, including diseases, but the molecular mechanisms through which the SNPs lead to phenotypic effects are often obscure. In this paper, we compiled a whole-genome catalogue of chromatin loci interacting with activating transcription factor 4 (ATF4), an important regulator of gene expression in cellular stress responses and other biological processes, and identified trait-associated SNPs overlapping ATF4 binding motifs. We then evaluated the consequences of more than 500 ATF4 binding motif-altering SNPs on allelic transcriptional activity using a massively parallel reporter assay in hepatoma cells in unstressed and stressful conditions. Further characterization of five selected SNPs in additional cellular models confirmed ATF4-dependent regulatory effects for the SNPs rs532446 (*GADD45A* intronic; linked to blood cell parameters), rs7011846 (*LPL* upstream; myocardial infarction), rs2718215 (diastolic blood pressure), rs281758 (psychiatric disorders) and rs6491544 (educational attainment). CRISPR/Cas9 technology was used to confirm the effect of rs532446 region on *GADD45A* and rs7011846 region on *LPL* gene expression. In conclusion, the data indicate that allelic variants of rs532446, rs7011846 and several other GWAS trait-linked SNPs located in ATF4 binding motifs differ in the ability to activate ATF4-mediated gene expression.

## Introduction

Activating Transcription Factor 4 (ATF4) is the principal regulator of gene expression during the integrated stress response (ISR) program, which is initiated when cells are exposed to various harmful conditions, such as nutrient deficiency, misfolded or unfolded protein overload in the endoplasmic reticulum, and oxidative stress [1–3]. In the absence of an overt stress condition, based on knockout mouse and other experimental systems, ATF4 has been implicated in a broad range of biological processes and homeostatic functions, including fetal hematopoiesis [4], megakaryocytopoiesis [5], skeletogenesis [6], eye development [7], memory formation [8], muscle atrophy [9] and carbohydrate and lipid metabolism [10]. The ATF4 protein is a bZIP transcription factor and binds to DNA as either a homodimer or a heterodimer with a variety of other bZIP transcription factors, such as C/EBP family members [2,11]. The chromatin binding sites of ATF4 in several cell types have been probed by ChIP-Seq experiments; however, detailed comparisons between the ATF4 binding profiles in different cell types has not been undertaken.

Genome-wide association studies (GWAS) have shown that trait-associated genetic variants tend to localize to regulatory elements, putatively acting through the perturbation of transcription factor binding sites [12]. Understanding the genetic variations within ATF4 binding sites could provide insights into the regulation of gene expression and the mechanisms underlying diseases or other traits, but this has not been studied systematically. Massively parallel reporter assay (MPRA) enables direct measurement of allelic differences in transcriptional regulation and, therefore, helps to identify affected functional transcription factor binding motifs [13]. Previously, MPRA has been used to identify susceptibility variants associated with many complex human diseases, including obesity [14], osteoarthritis [15], type 2 diabetes [16] and melanoma [17]. In the current article, we carried out MPRA of chromatin regions containing trait-linked SNPs located within ATF4 binding sites and compared the transcriptional activity of the individual alleles. Further, ATF4 ability to bind to the allelic variants of selected SNPs and activate transcription was validated by EMSA and conventional luciferase assays, and CRISPR/Cas9 technology was used to confirm the regulatory effect of rs532446 (associated to platelet and red blood cell characteristics) on *GADD45A* and rs7011846 (associated to myocardial infarction) on *LPL* gene expression in the endogenous genomic context.

## Results

### Catalog of ATF4 binding sites in the human genome

**Fig 1:**
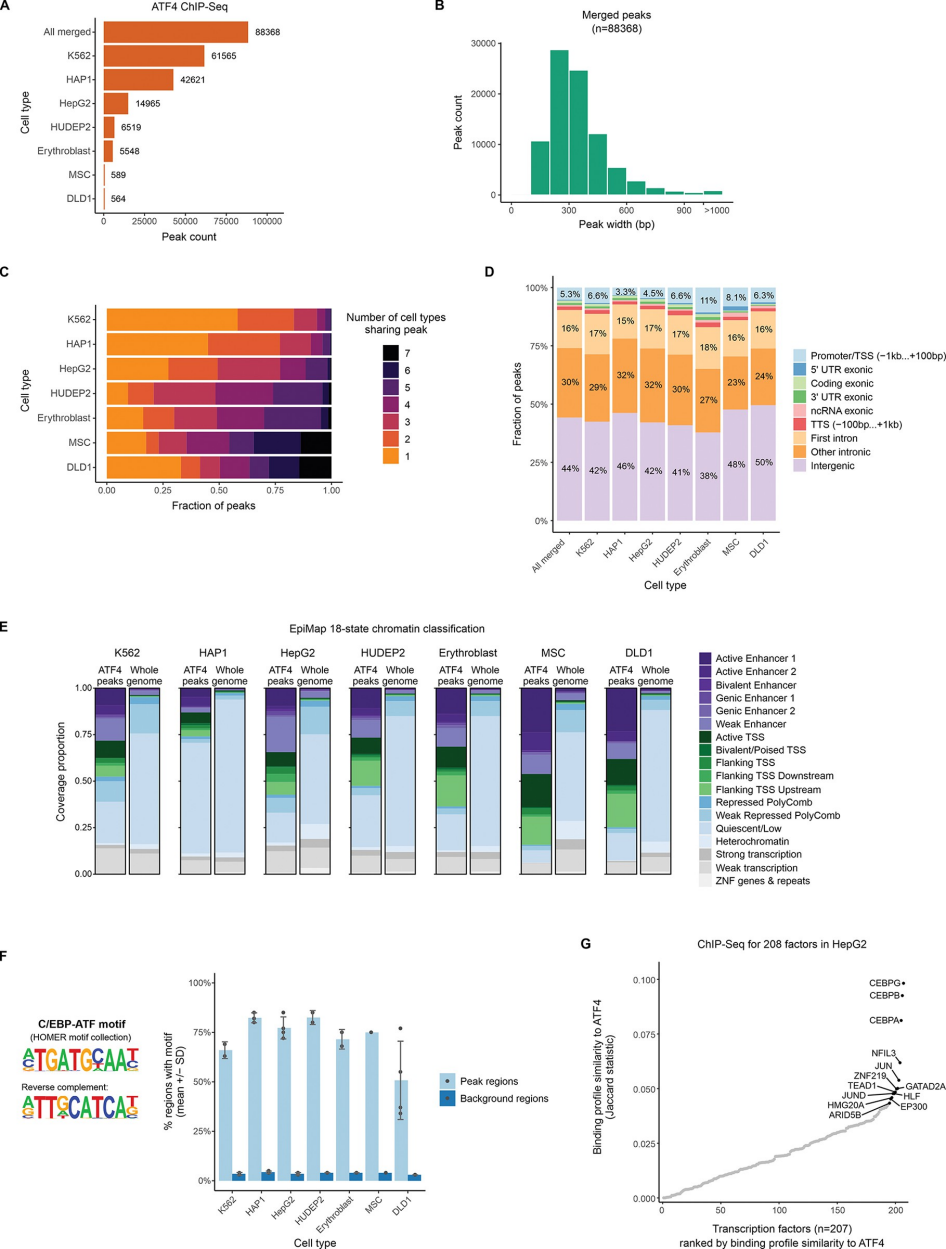
Systematic analysis of ATF4 binding sites in the human genome. **(A)** Number of peaks called in ATF4 ChIP-Seq experiments in human cell types. Data from published experiments was reprocessed from raw reads using a uniform pipeline. To obtain a common peak set across all cell types, overlapping peak regions were merged. **(B)** Peak length distribution for the merged set of ATF4 peak regions. **(C)** Fraction of ATF4 ChIP-Seq peaks called in more than one cell type. **(D)** Genomic context distribution of ATF4 peaks. **(E)** Functional state of chromatin in ATF4 ChIP-Seq peak regions and genome-wide. Epigenomic maps were obtained from the EpiMap project [25], which annotated chromatin into 18 states based on combinations of histone modifications. For HepG2, K562, HAP1 and MSC, basal (non-stress condition) data was available in EpiMap (accessions BSS00558, BSS00762, BSS00491 and BSS01260, respectively). For erythroblast, HUDEP2 and DLD1, a similar EpiMap cell type was used as a proxy (erythroid differentiation of CD34+ cells for the former two, and immortalized colonic epithelial cells for the latter; EpiMap accessions BSS00545 and BSS00223, respectively). **(F)** Percentage of peak regions containing the C/EBP-ATF motif, a known ATF4 binding sequence, compared to matched background regions (as selected by HOMER). The peak summit coordinate +/- 100 bp was used as the search region. The HOMER motif database ATF4 motif (based on GSE35681) was used as a representative C/EBP-ATF motif. Each dot represents a ChIP-Seq library. **(G)** ChIP-Seq binding profile similarity between ATF4 and 207 other chromatin-binding proteins in HepG2 cells. The peak sets besides ATF4 were obtained from [27] (GEO accession: GSE104247). For ATF4, peaks from all HepG2 ChIP-Seq samples were merged. All peaks were resized to equal length (+/- 100 bp around the center) and the bedtools Jaccard similarity statistic [28] was calculated between the ATF4 binding regions and every other set of ChIP-Seq peaks.

To assemble a collection of human ATF4 binding sites, we gathered raw ChIP-Seq data from 18 previously published ATF4 immunoprecipitation libraries spanning 7 cell lines across diverse cellular lineages and 4 different antibodies (S1 Table) [18–24]. The raw read data was reprocessed using a uniform pipeline and genome build (GRCh38) as detailed in the Materials and Methods. All libraries were of satisfactory quality based on the statistics of read alignment and peak calling (S2 and S3 Tables). Merging overlapping peaks by cell line resulted in 564 to 61,565 peaks per cell line (Fig 1A), and merging peaks across all cell lines resulted in an overall set of 88,368 peaks with a typical size of around 300 bp (Fig 1B, S4 Table). In all cell lines except K562 (which had the most peaks called), more than half of the peaks were also detected with at least one other cell line (Fig 1C). Cell lines with less overall peaks tended to have a high fraction of shared peaks (Fig 1C), lending support to the validity of those peak calls.

The genomic context of the ATF4 peaks showed a similar trend in all 7 cell lines, with intergenic and intronic regions harboring the largest fraction of peaks, and with generally less than 10% (maximum 11%) of peaks localizing to promoter regions (Fig 1D, S4 Table). To investigate the type of chromatin prevalent at ATF4 binding sites, we used the EpiMap 18-state model of genome epigenetic state [25]. As shown in Fig 1E, in all 7 cell lines, ATF4 peak regions were enriched for active chromatin, such as enhancers and promoter-flanking regions, relative to the rest of the genome, which was mostly classified as quiescent chromatin.

ATF4 is known to be a principal activator of transcription from the C/EBP-ATF composite motif [26]; thus, we sought to confirm the occurrence of this motif in the ATF4 ChIP-Seq peaks to confirm signal specificity. Across all cell lines and libraries, ATF4 peak regions consistently demonstrated strong enrichment of the C/EBP-ATF motif defined in the HOMER motif catalog, with an average of 70.9% of peaks harboring a C/EBP-ATF motif, compared to 3.7% of background regions (Fig 1F, S2 and S5 Tables). In *de novo* motif analysis, the most enriched motif for every library was highly similar and corresponded to a C/EBP-ATF sequence (S1 Fig). For HepG2 cells, ATF4 ChIP-Seq libraries were available from both stressed (bortezomib-induced ER stress) and untreated cells (S1 Table). Comparing peak sets for the treatments revealed that the strongest peaks of each condition tend to be ones that were also detected in the other condition (S2A, S2B Fig) and that the preferred binding motif was nearly unchanged by treatment (S2C Fig), suggesting a similar binding profile with mostly quantitative changes due to increased protein abundance in stress. For HAP1 cells, ATF4 ChIP-Seq was performed with three different anti-ATF4 antibodies in the same study [18] (S1 Table). Comparing the peak sets revealed that the majority of peaks for each antibody were shared with other antibodies (S3A Fig), with the more confidently called peaks being more likely shared with other antibodies (S3B Fig). Further, the most enriched motif is highly similar for all three antibodies (S3C Fig), indicating similar specificity.

Besides the C/EBP-ATF motif, additional enriched motifs included (depending on the library) motifs corresponding to AP-1, CREB and C/EBP families (S6 Table). To identify TFs with the most similar binding profiles to ATF4, we utilized ChIP-Seq data for 208 chromatin binding factors in HepG2 [27]. Using the bedtools Jaccard statistic [28] as a similarity metric, ATF4 demonstrated the highest binding profile similarity towards several C/EBP and AP-1 family members (Fig 1G), in line with the results from *de novo* motif enrichment.

### ATF4 downstream effects based on chromatin binding and RNA profiling studies

**Fig 2:**
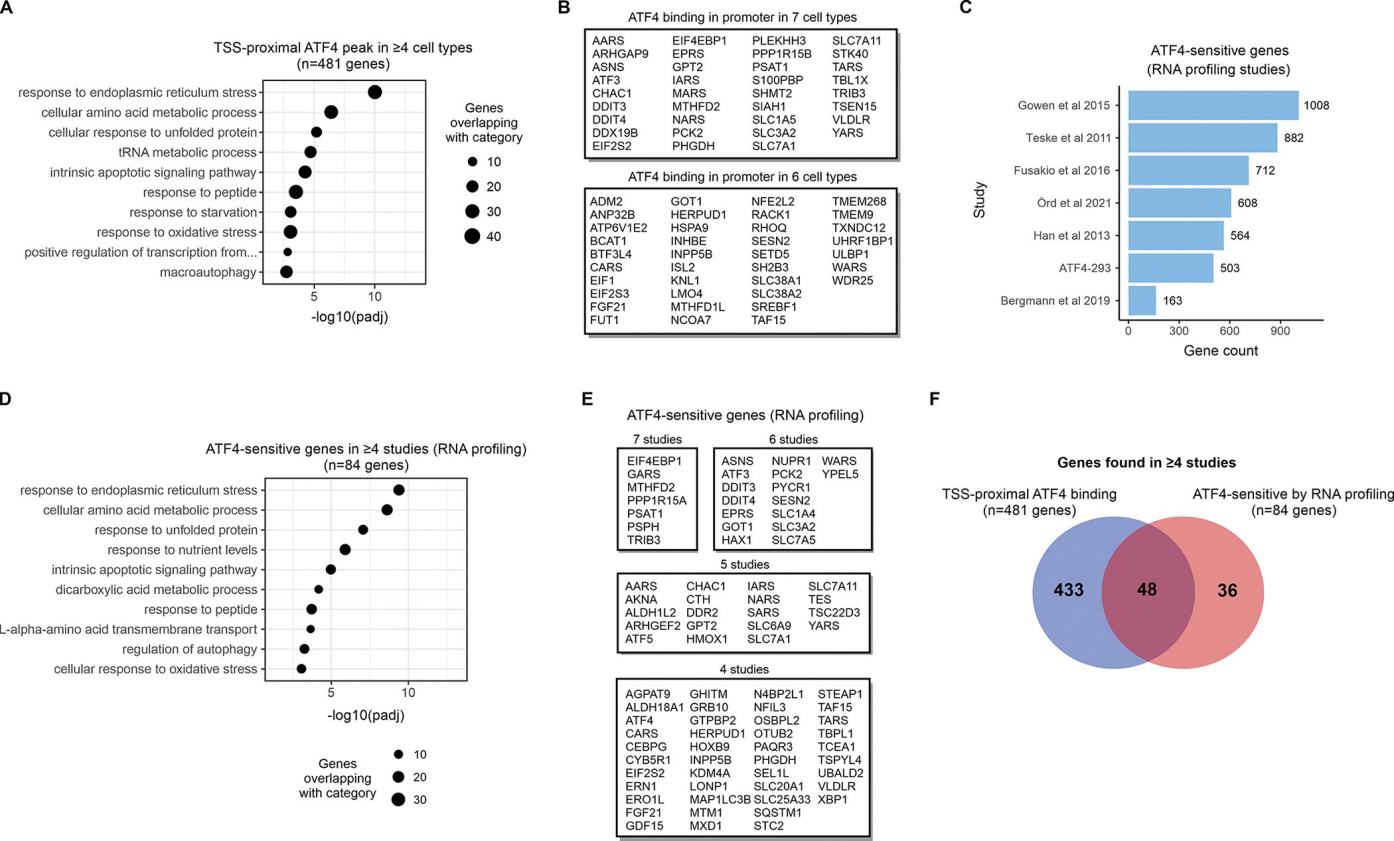
ATF4-sensitive genes and biological pathways. **(A)** Gene ontology biological process enrichment analysis for genes with a TSS-proximal ATF4 peak called in ≥4 cell types (out of the 7 analyzed). One ChIP-Seq library was used to represent each cell type (the library with the most peaks called). **(B)** Genes most frequently binding ATF4 at the TSS/promoter. All genes with binding in 6 or 7 cell types are listed. **(C)** Number of ATF4-sensitive genes from RNA profiling studies of ATF4 pathway modulation. **(D)** Gene ontology biological process enrichment analysis for genes with ATF4-sensitive expression in ≥4 studies (out of the 7 analyzed). **(E)** Most frequently ATF-sensitive genes across the RNA profiling studies. **(F)** Intersection of genes with ATF4 binding at the TSS/promoter region in ≥4 ChIP-Seq studies and ATF4-sensitive expression in ≥4 RNA profiling studies.

To relate ATF4 chromatin binding patterns to biological functions, we looked at ATF4 binding sites that are near gene promoters (marked in S3 Table), as in these cases there is a clear candidate for the target gene. In total, 481 genes displayed ATF4 binding at the promoter in ≥4 cell lines, and, in line with the known cellular roles of ATF4 [1,29], this set of genes was most significantly enriched for processes related to endoplasmic reticulum stress, amino acid metabolism, oxidative stress and apoptosis (Fig 2A). When analyzed individually, all 7 cell lines displayed enrichment for largely these same biological processes (S4A Fig), and the enrichment results also remained consistent when the gene sharing criterium was varied from ≥3 up to all 7 cell lines (S4B Fig), indicating a consistent core functionality for ATF4 across these cell types. The most genes frequently displaying ATF4 binding at the promoter, in either 6 or all 7 cell lines, are listed in Fig 2B, and include several previously characterized ATF4-regulated genes such as *ASNS*, *DDIT3* (*CHOP*) and *TRIB3* [2].

To associate distal ATF4 binding events to potential target genes, we utilized data from the activity-by-contact (ABC) model [30], a method that predicts enhancer-gene connections based on chromatin interaction and enhancer activity data. Of the cell types with ATF4 ChIP-Seq, ABC maps were available for HepG2, K562, HAP1 and erythroblast, considerably expanding the set of potential target genes (S7 Table). Genes linked to distal ATF4 peaks via the ABC model were enriched for similar functional categories as the genes with ATF4 binding in the promoter (described above), though the enrichment was milder for the distal elements, possibly suggesting that distally bound ATF4 have more diverse functions (S4C and S4D Fig).

To gauge the output of ATF4 activity on genome-wide gene expression, we collected data from RNA profiling studies where ATF4 was modulated in different cell and tissue contexts by means of knockout, overexpression or chemical perturbation (S8 Table) [18,21,31–34]. In addition to 6 published sources (including one previous meta-analysis [31]), we included a novel set of gene expression profiles from HEK293-derived ATF4-293 cells, where ATF4 is under the control of a tetracycline-activated promoter. Gathering the ATF4-sensitive genes (in the positively regulated direction) resulted in several hundred genes for most data sources (Fig 2C, S8 Table). Gene ontology enrichment analysis for genes frequently (≥4 studies) displaying ATF4-sensitive expression level revealed biological processes highly similar to the enrichment analysis of genes with promoter-bound ATF4 (Figs 2A, 2D, S4B, S5A). A number of genes occurred frequently in the data sets (including 7 genes present in all 7 data sources) and these recurrent genes may represent core ATF4 downstream genes shared across diverse cell types (Fig 2E). At the level of individual studies however, there was some variability in the biological processes associated with ATF4-sensitive genes; notably, lipid metabolism genes displayed positive regulation by ATF4 in the liver (S5B Fig) [33]. Intersecting ATF4-sensitive genes from RNA profiling studies (84 genes found in ≥4 studies) with genes frequently displaying ATF4 binding in the promoter (481 genes found in ≥4 studies) revealed that 57% of these ‘core’ ATF4-sensitive genes also show promoter-bound ATF4 (Fig 2F). The non-overlapping genes may represent effects of ATF4 bound to distal regulatory elements, indirect regulatory effects (such as other TFs acting downstream of ATF4), and/or mismatch between the experimental contexts of the DNA and RNA studies.

### Massively parallel functional profiling of human genetic variation affecting ATF4 binding sites

To assess how human natural genetic variation might affect ATF4 binding sites, we intersected the merged set of ATF4 ChIP-Seq peaks (n = 88,368; Fig 1A) with all common SNPs and short indels (MAF > 0.01 in at least one reference population) and retained all genetic variants that overlapped (with one or both alleles) an ATF4-associated DNA motif. To define the motif overlaps, we considered motifs as 10-mers with N-s in the lenient nucleotide positions, since for the related factor C/EBPβ it has been reported that the positions immediately flanking the core 8-mer act as important modifier nucleotides [35]. Thus, the search was aimed at finding motif-altering variants in general, including cases that are not necessarily predicted to be completely motif-disrupting. We considered the C/EBP-ATF motif (NTGATGNAAN; Fig 1F), the closely related CREB-C/EBP motif (NTGACGNAAN), also reported to bind ATF4 heterodimers [36,37], the CRE motif (NTGACGTCAN), bound by several favorable bZIP heterodimers of ATF4 and an AP-1 family member [11] and the BATF-ATF motif (NTGACGTGNC), bound by favorable bZIP dimers such as BATF3-ATF4 and BATF2-ATF4 [11]. This resulted in 9,579 genetic variants (S9 Table), with C/EBP-ATF matches being the most frequent (8,305 variants), followed by CREB-C/EBP (2,336; variants may overlap multiple motifs). To confirm that this consensus-based strategy was not leading to a loss of variants, we compared it to position weight matrix (PWM) scoring using the most significant *de novo* motif found in ATF4 peaks, which resembles a C/EBP-ATF site and was found in 67.25% of peaks and 1.73% of background regions (S6A Fig, S6 Table). HOMER PWM scoring for this motif nominated slightly fewer (7,633) variants as overlapping the motif (with at least one allele) than the consensus-based method (8,305 C/EBP-ATF overlaps; S6B Fig). Out of the variants identified by HOMER, 7,152 (93.7%) were also found by the C/EBP-ATF consensus, and a further 432 (5.7%) were found by the CREB-C/EBP consensus (S6B Fig). The remaining 49 (0.6%) variants that were only nominated by HOMER PWM scoring all ranked among the bottom 10% by their HOMER motif match score (the highest-scoring allele was used for each variant; S6C Fig).

To test whether the genetic variants overlapping ATF4 peaks and motifs are enriched for transcriptional regulatory variants, we intersected them with statistically fine-mapped (likely-causal) eQTL variants from GTEx v8 [38]. Variants localized to ATF4 peaks were enriched for eQTL variants to a similar degree (approximately 2-fold) as variants localized in enhancers, which are defined by independent means (S10 Table). If the annotations are combined (variant in enhancer with ATF4 peak and motif overlap), the enrichment strengthened to 2.7-fold (S10 Table). Similarly, promoter and 5′-UTR variants in general were enriched for eQTL variants by 6.6- and 7.2-fold, respectively, which increased to 10.0 and 17.8-fold enrichment, respectively, when the variant was additionally overlapping an ATF4 motif in an ATF4 peak (S10 Table).

To assess which of the ATF4 motif-overlapping variants have evidence for affecting human traits, we used the OpenTargets Genetics portal [39,40], which aggregates GWAS and PheWAS data and performs LD expansion to identify proxy SNPs for the association lead SNPs. In total, 581 motif-altering variants showed association to at least one human trait, either as lead or proxy SNP (S11 Table). In many cases, the allele frequencies of these SNPs varied considerably between human populations. Among the six populations of the gnomAD v2.1 whole-genome dataset [41], for 71 SNPs the alternative allele was common (allele frequency >0.05) in at least one population while being rare (<0.01) in at least one other (S11 Table). Relative to the minor allele frequency in non-Finnish Europeans, some other population showed at least a 5-fold increase or decrease in allele frequency for 46 and 141 SNPs, respectively (S11 Table). To prioritize the target genes for these variants, we noted any nearby TSS-s, whether any of these nearby genes displayed ATF4-sensitive expression, and also the predicted target genes from the OpenTargets Variant-to-Gene pipeline [39,40], the ABC model [30] and GTEx [38] (S12 Table).

**Fig 3:**
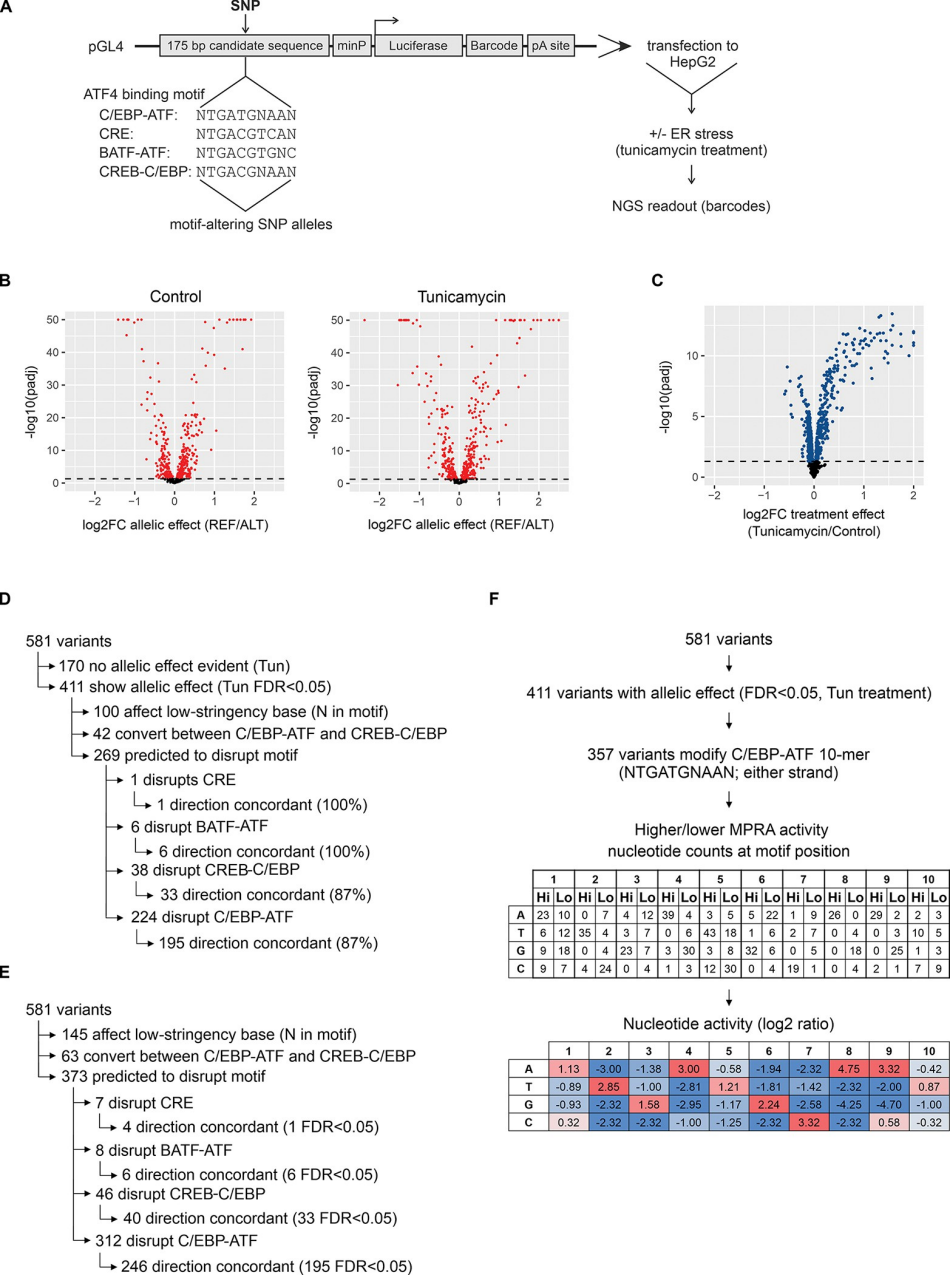
MPRA of human genetic variation affecting ATF4 binding sites. **(A)** Schematic overview of the MPRA experiment. In the reporter plasmid, each insert (SNP-containing fragment) is associated with specific 10-nucleotide barcodes (6 different barcodes per fragment and allele) which will be incorporated into the transcript and used as the assay readout. **(B)** Allelic effect results of MPRA in Control or tunicamycin-treated HepG2 cells (n = 5 experiments per condition). Genetic variant allelic effects (n = 581 variants) were analyzed using MPRAnalyze. Fold change (FC) is calculated as the reference allele relative to the alternative allele. **(C)** Effect of tunicamycin treatment on MPRA reporter activity. All variant regions (n = 581) are represented with both reference and alternative alleles (total 1162 sequences) and were tested for treatment effect using voom-limma. **(D)** Concordance between MPRA allelic effect and the effect predicted from motif disruption. All significant (FDR<0.05) MPRA allelic effect results from tunicamycin treatment were included. **(E)** Validation rate of predicted motif disruptions in the MPRA experiment. All SNPs predicted to disrupt a motif were included. **(F)** Evaluation of the preferred (higher-activity) nucleotide at each position of the C/EBP-ATF 10-mer motif according to allelic MPRA results. All SNP allelic comparisons (pairwise comparisons of REF and ALT) that showed a significant allelic activity difference (FDR<0.05 in tunicamycin treatment) were included. For each SNP, the position of the SNP within the motif 10-mer was determined, with the motif always in the same strand orientation (NTGATGNAAN). Then, based on the allelic effect direction in the MPRA, the SNP nucleotides were added to either the higher (Hi) or lower (Lo) activity nucleotide count for their respective position in the motif. For each motif position, the log2 ratio for Hi activity over Lo was calculated for each nucleotide, adding a pseudocount of 1. In panels A and B, P values were adjusted using FDR and the dashed line indicates FDR 0.05.

To test the 581 motif-altering, trait-associated SNPs for an allelic regulatory effect, the SNP alleles, flanked by 87 bp on both sides, were inserted in the MPRA vector plasmid described previously [42] (Fig 3A). Each SNP allele was synthesized associated to 6 randomly assigned barcodes used as the readout (S13 Table), and we also introduced UMI-s into the protocol to correct for potential PCR duplicate reads. To find the optimal cell culture conditions, HepG2 human hepatoma cells were transfected with the MPRA library and exposed to the ER stress inductor tunicamycin to activate ATF4. The MPRA vector backbone carries the luciferase ORF (Fig 3A), and the bulk luciferase activity of the library (presumably driven by the high incidence of ATF4 motifs) showed upregulation after 18 h exposure to 2 μg/ml tunicamycin (S7A Fig). At the same time, cell viability was only slightly reduced; thus, these conditions were chosen for the MPRA experiment (S7B Fig). Performing 5 independent experiments (transfections done on separate days) in HepG2 cells (with and without tunicamycin-induced ER stress) resulted in sequencing data showing a strong correlation between replicates at the level of raw counts (S7C Fig) as well as normalized MPRA activity (RNA signal normalized to DNA input) (S7D Fig.). As positive controls, the library contained regions with previously validated ATF4 response elements from 8 genes (S8A Fig.) as well as viral enhancer sequences and TF motif multimers (S13 Table; S8B Fig). While most known ATF4 response elements are promoter-localized C/EBP-ATF sites, the *PPP1R15A* (*GADD34*) promoter uses a CRE motif [43] and the *SARS1* gene uses an intronic C/EBP-ATF [44]. Substitutions introduced to disrupt the known ATF4 response elements resulted in lower MPRA activity compared to the wild type sequences, as expected (S8A Fig, S14 Table).

For statistical testing of SNP allelic effects, we employed the MPRAnalyze package [45], which is designed to analyze barcode-based MPRA data, where the same candidate DNA sequence is associated with multiple readout barcodes, and where measures are ratios of RNA to DNA. In control and tunicamycin-treated HepG2 cells, 359 and 411 SNPs, respectively, showed a significant allelic effect (FDR<0.05) (Fig 3B, S15 Table), with most variants shared and directionally concordant between the treatments (S9A Fig). Tunicamycin treatment was more likely to upregulate than downregulate the candidate sequences in this library, as expected (Figs 3C, S7A), and allelic effect sizes tended to increase under tunicamycin treatment (S9B Fig). Whether the variant was localized to a promoter or distal region in the genome did not appear to affect the detection frequency (S9C Fig) or effect size (S9D Fig) of the allelic effects. To evaluate the allelic effect statistical analysis, we focused on directional concordance between effect direction measured in MPRA and that predicted from the motif. Based on previously studies of *Atf4* knock-out mouse cells and other models, ATF4 appears to be a positive regulator of transcription [26,32]. In tunicamycin treatment, there were 269 variants for which MPRA detected an allelic effect (at FDR<0.05) and the SNP was predicted to disrupt a motif (that is, alter a non-N base in the motif; S15 Table). In ≥87% of cases, the MPRA effect was directionally concordant with that predicted from the motif sequence (Fig 3D). In turn, we evaluated how likely a motif disruption predicted from sequence would result in an MPRA allelic effect (Fig 3E). Out of 373 SNPs predicted to disrupt a motif, approximately two thirds produced in a directionally concordant and statistically significant MPRA result (Fig 3E). For a small fraction (13%) of allelic effects, motif disruption was not directionally concordant with measured allelic activity (S15 Table; S9E, S9F Fig). This motif-discordant fraction, totaling 38 variants across the treatments, did not appear to be explained by variant location (promoter/distal; S9E Fig) or the type of ATF4 motif disrupted (S9F Fig), and the mechanisms behind these effects remain unknown. To evaluate whether MPRA revealed preference at the non-stringent positions of the C/EBP-ATF motif, SNP nucleotides were classified as higher or lower activity based on their MPRA result and the nucleotide counts summarized by their position in the 10-mer motif. This revealed ATGATGCAAT as the preferred nucleotide sequence (Fig 3F), which matches the trend seen in the ChIP-Seq based motifs (S1 Fig), indicating that ATF4 transcriptional output is in line with the binding preference seen in ChIP-Seq even at the flanks of the 10-mer motif.

### Confirmation of ATF4-mediated regulatory effect for selected GWAS SNPs

**Fig 4:**
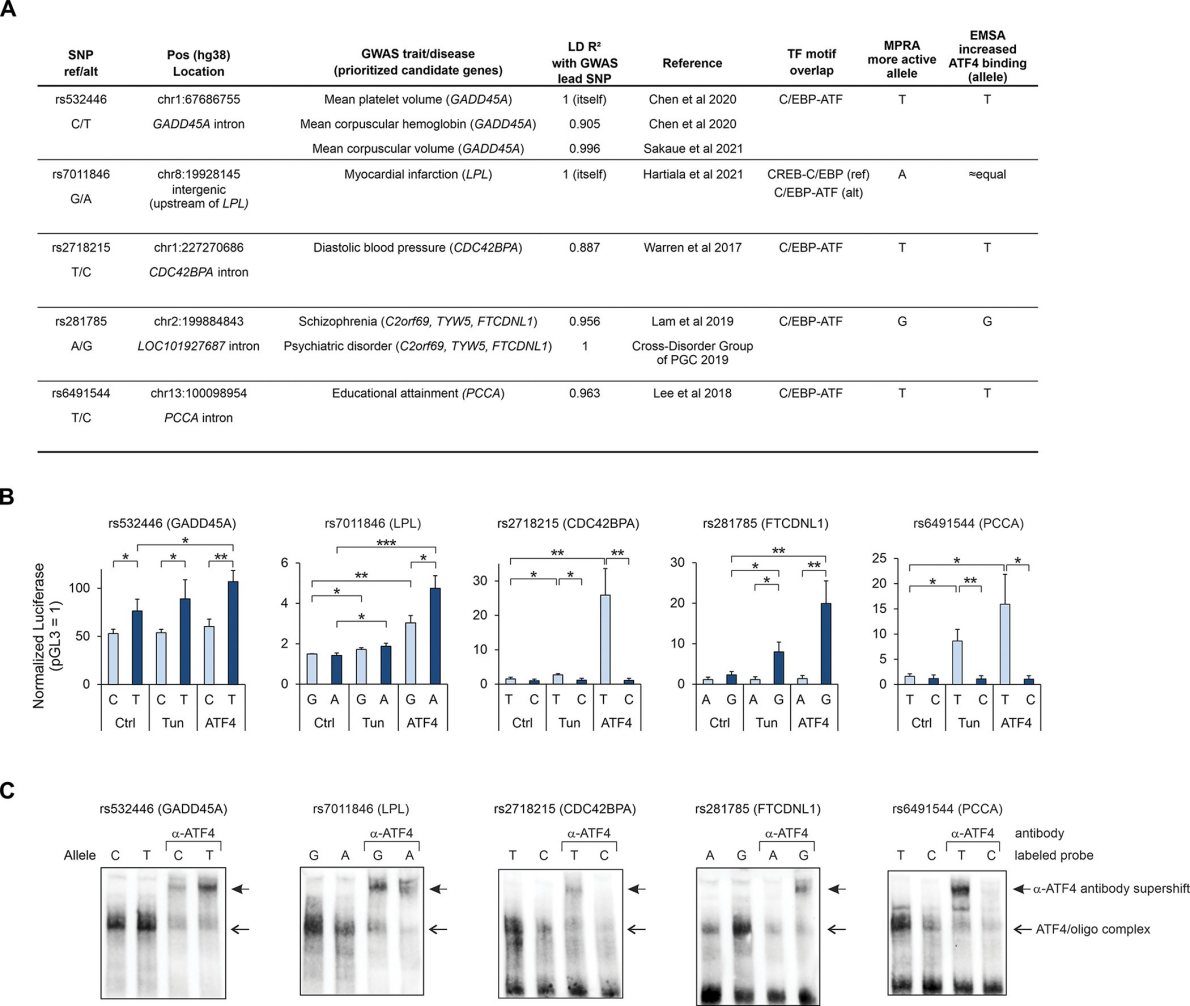
Characterization of selected SNPs. **(A)** Overview of SNPs selected for validation of ATF4-dependent allelic effect. Genetic association data was queried from OpenTargets. **(B)** Luciferase reporter activity for genomic regions containing SNP alleles (ref, light blue; alt, dark blue) in HepG2 cells without treatment (Ctrl), with tunicamycin treatment (Tun) and with ATF4 overexpression (ATF4). The mean ± SD is shown from 4–5 experiments performed on separate days. **(C)** Electrophoretic mobility shift assay (EMSA) with biotin-labeled oligonucleotides corresponding to either the ref or the alt allele of the SNP. Nuclear protein extract from HepG2 cells overexpressing ATF4 was used, and, in lanes labeled α-ATF4, antibody targeting ATF4 was included in the reaction. *p < 0.05, **p < 0.005, ***p < 0.0005 by two-tailed *t* test with Bonferroni-Holm correction.

For further molecular characterization, we selected 5 genetic variants where high LD between the GWAS lead SNP and the MPRA SNP indicated good potential for the GWAS SNP to act through the modification of the ATF4 binding site (Fig 4A). To validate the MPRA results, we conducted luciferase reporter assays for the genomic regions, comparing the reference and alternative alleles. To confirm that ATF4 mediates the regulatory effects of the SNPs, we performed the reporter assay using ATF4 overexpression and additionally carried out the electrophoretic mobility shift assay (EMSA), including supershift experiments with anti-ATF4 antibody.

A region demonstrating strong enhancer activity in our MPRA was the fragment containing SNP rs532446 (chr1:67,686,755 C/T), which resides in an intron of the *GADD45A* gene (1,554 bp from the TSS). The SNP rs532446 is associated with several GWAS phenotypes related to the megakaryocyte-erythroid lineage of blood cells [46,47] (Fig 4A, S11 Table), and GADD45A has been implicated in hematopoietic stem cell differentiation in mouse studies [48]. The frequency of the alternative allele (T) is 0.40 in the African/African-American population but more frequent (0.61–0.73) in the other populations of gnomAD v2.1 [41] (S11 Table). The variant overlaps a low-stringency position within a C/EBP-ATF motif (GTTTCATCA[C/T]). In agreement with this, both alleles exhibited transcriptional activity in a luciferase assay in untreated, tunicamycin-treated or ATF4-overexpressing cells; however, the activity of alternative allele was significantly higher than that of the reference allele (Fig 4B), in accordance with the MPRA result (S15 Table). Further, EMSA of HepG2 (Fig 4C) and ATF4-293 cells (S10A and S10B Fig) indicated that ATF4 binds more strongly to the alternative allele. This effect direction is in line with eQTL data, where the alternative allele is associated with increased *GADD45A* mRNA expression (TwinsUK, lymphoblastoid cells, p = 6.2e-9; effect size 0.15) [49].

The SNPs rs2718215 (intronic to *CDC42BPA*; blood pressure [50]; A[T/C]TTCATCAC), rs6491544 (intronic to *PCCA*; educational attainment [51]; A[T/C]GATGAAAT) and rs281785 (intronic to *LOC101927687*; psychiatric disorders [52,53]; ATGAT[A/G]CAAC) each overlap C/EBP-ATF motifs in high-stringency positions, where one allele could be considered a motif disruption (Fig 4A, S11 Table). In line with this, luciferase assays for these SNPs demonstrated large allelic effects (Fig 4B) which were directionally concordant with the predicted motif disruption and with the MPRA results (S15 Table). In luciferase experiments, the motif-intact alleles of these SNPs were responsive to tunicamycin-induced ER stress and ATF4 overexpression (Fig 4B). For all three SNPs, EMSA confirmed preferential ATF4 binding at the higher-activity allele in HepG2 (Fig 4C) and ATF4-293 cells (S10A, S10B Fig). eQTL data is directionally concordant with our functional experiments and suggests *CDC42BPA* as the target gene for rs2718215, *PCCA* for rs6491544, and *TYW5* and *FTCDNL1* for rs281785 [54].

The SNP rs7011846 (intergenic, upstream of *LPL*; ATTGC[G/A]TCAC) is a GWAS lead SNP for myocardial infarction (MI) [55] and resides in a macrophage- and adipose-specific open chromatin region [56]. The frequency of the alternative allele (A) is highly variable between populations, from 0.003 in East Asian to 0.279 in African/African-American, with Non-Finnish European frequency in between (0.026) [41] (S11 Table). The SNP causes a switch between CREB-C/EBP (reference allele) and C/EBP-ATF (alternative allele) motifs, both of which are reported ATF4 binding motifs (Fig 4A). In MPRA, the alternative allele demonstrated higher activity in both control and tunicamycin conditions (S14 and S15 Tables). In luciferase, both alleles appeared equal under these treatments; however, higher activity of the alternative allele was evident when ATF4 was overexpressed (Fig 4B). In EMSA in two cell lines, both alleles of rs7011846 appeared to bind ATF4 (Figs 4C and S10); thus, it is possible that the switch between CREB-C/EBP and C/EBP-ATF motifs causes a change in the preferred heterodimerization partner(s) of ATF4, leading to enhanced transcriptional activity from alternative allele (with the C/EBP-ATF site) in certain settings.

### The SNP rs532446 located in the third intron of *GADD45A* regulates the stress-induced expression of the gene in K562 cells

**Fig 5:**
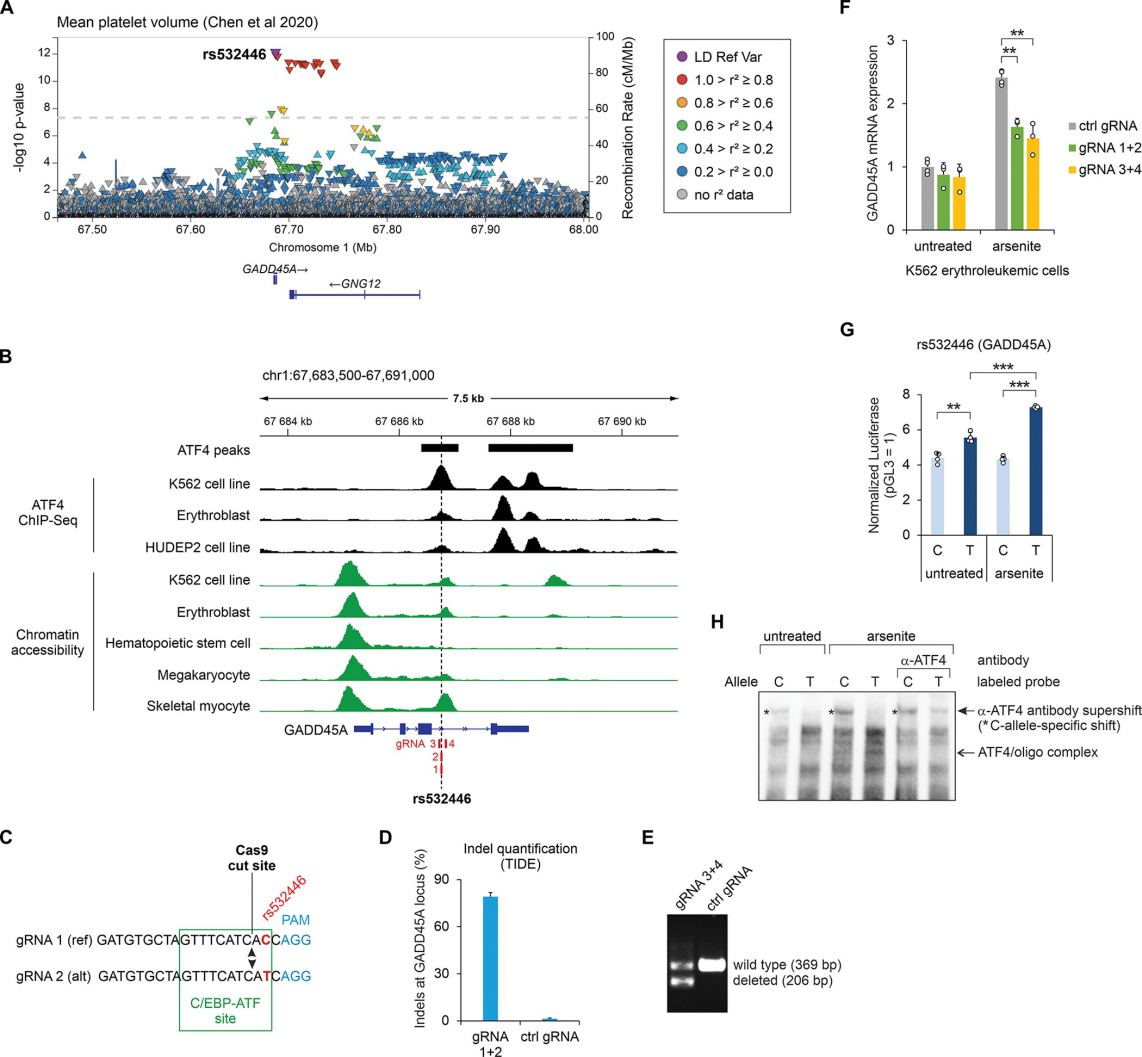
Effect of the rs532446-harboring regulatory region on *GADD45A* expression in K562 cells. **(A)** LocusZoom plot of the mean platelet volume GWAS study conducted by Chen et al [46]. The trait association *p* values for all variants studied in this region are shown and the orientation of the arrowheads indicates the effect direction (GWAS Catalog accession: GCST90002346). Linkage disequilibrium (LD) in the European population is shown relative to the lead SNP (rs532446), provided by LocusZoom [98]. **(B)** Chromatin accessibility and ATF4 ChIP-Seq coverage near rs532446. The SNP location is indicated with a vertical dashed line, and the locations of CRISPR gRNAs are shown in red. Chromatin accessibility tracks were obtained from the human single-nucleus ATAC-Seq atlas [56], except K562, which was obtained from ENCODE accession ENCFF972GVB [99]. **(C)** CRISPR gRNAs designed to introduce short indels within the C/EBP-ATF motif overlapping rs532446. The gRNA sequences targeting the reference (ref) and alternative (alt) alleles are shown, and the locations of the C/EBP-ATF motif (green), the rs532446 SNP (red), the gRNA PAM motif (blue), and the Cas9 cut site (arrowheads) are indicated. **(D)** Quantification of indels introduced into the rs532446-overlapping C/EBP-ATF motif by the CRISPR-Cas9 editing using gRNA 1+2 (described in panel C). **(E)** PCR-based validation of CRISPR-Cas9 mediated deletion of the rs532446 region using gRNA 3+4. **(F)** RT-qPCR quantification of *GADD45A* mRNA expression in untreated and arsenite-treated cells transfected with control gRNA (ctrl gRNA) or gRNAs designed to disrupt the regulatory region overlapping rs532446 (gRNA 1+2 or gRNA 3+4). Data are presented relative to the mRNA expression level in untreated cells transfected with ctrl gRNA. **(G)** Luciferase reporter activity for genomic regions containing SNP rs532446 (C/T) in untreated and arsenite-treated K562 cells. **(H)** EMSA analysis for SNP rs532446 allele-specific binding of ATF4. Biotin-labeled probes containing rs532446 C or T alleles were incubated with nuclear extracts from untreated and arsenite-treated K562 cells. In lanes labeled α-ATF4, antibody targeting ATF4 was included in the reaction. In panels F and G the mean ± SD is shown from 3–4 transfection experiments performed on separate days. **p < 0.005, ***p < 0.0005 by two-tailed *t* test with Bonferroni-Holm correction. The coordinates shown are for GRCh38.

The SNP rs532446 is associated to several GWAS phenotypes related to the platelets and red blood cells (4A, 5A). ATF4 ChIP-Seq data from erythroblast, K562 and HUDEP2 cells shows that ATF4 binds to the third intron of *GADD45A*, in the region containing rs532446, and this region overlaps with the chromatin accessibility peak in several cell types (Fig 5B). To elucidate the role of rs532446 in the regulation of *GADD45A* expression, two approaches to disrupt the rs532446 locus in K562 cells by CRISPR-Cas9 were carried out. First, the cells were transfected with SNP-overlapping gRNAs that direct Cas9 nuclease to cleave DNA within the C/EBP-ATF site, one nucleotide besides the SNP (Fig 5C), damaging the C/EBP-ATF motif. Since K562 cells are heterozygous for rs532446, a mixture of two gRNAs (1+2) was used, one specific to SNP reference allele and the other to the alternative allele. Sanger sequencing trace analysis [57] (TIDE method) revealed that Cas9 nuclease activity had introduced short indels in more than 80% of DNA molecules (Fig 5D). As a parallel approach, a mixture of two gRNAs (3+4) was used to delete a 163-bp fragment encompassing the SNP rs532446 from the K562 genome (Fig 5B, 5E). The cells edited with the CRISPR-Cas9 were exposed to arsenite, an inducer of the ATF4 ISR program known to activate *GADD45A* transcription [58], or left untreated. As presented in Fig 5F, arsenite treatment upregulated the expression of *GADD45A* in cells transfected with control gRNA, and the induction was significantly reduced in the cells transfected with the mixture of gRNA 1+2 as well as with the mixture of gRNA 3+4. In untreated cells, the expression of *GADD45A* was not affected by the disruption of rs532446 locus (Fig 5F). Furthermore, in a luciferase reporter assay in K562 cells, the rs532446 alternative (T) allele demonstrated significantly higher transcriptional activity and inducibility by arsenite stress, compared to the reference (C) allele (Fig 5G). EMSA using K562 cells exposed to arsenite revealed that ATF4 binds more strongly to the rs532446 T allele than to C allele (Fig 5H). Thus, the results indicate that the SNP rs532446 is involved in the regulation of *GADD45A* expression during the ISR.

### The myocardial infarction-associated SNP rs7011846 region affects the basal and thapsigargin-induced expression of *LPL* in THP-1 cells

**Fig 6:**
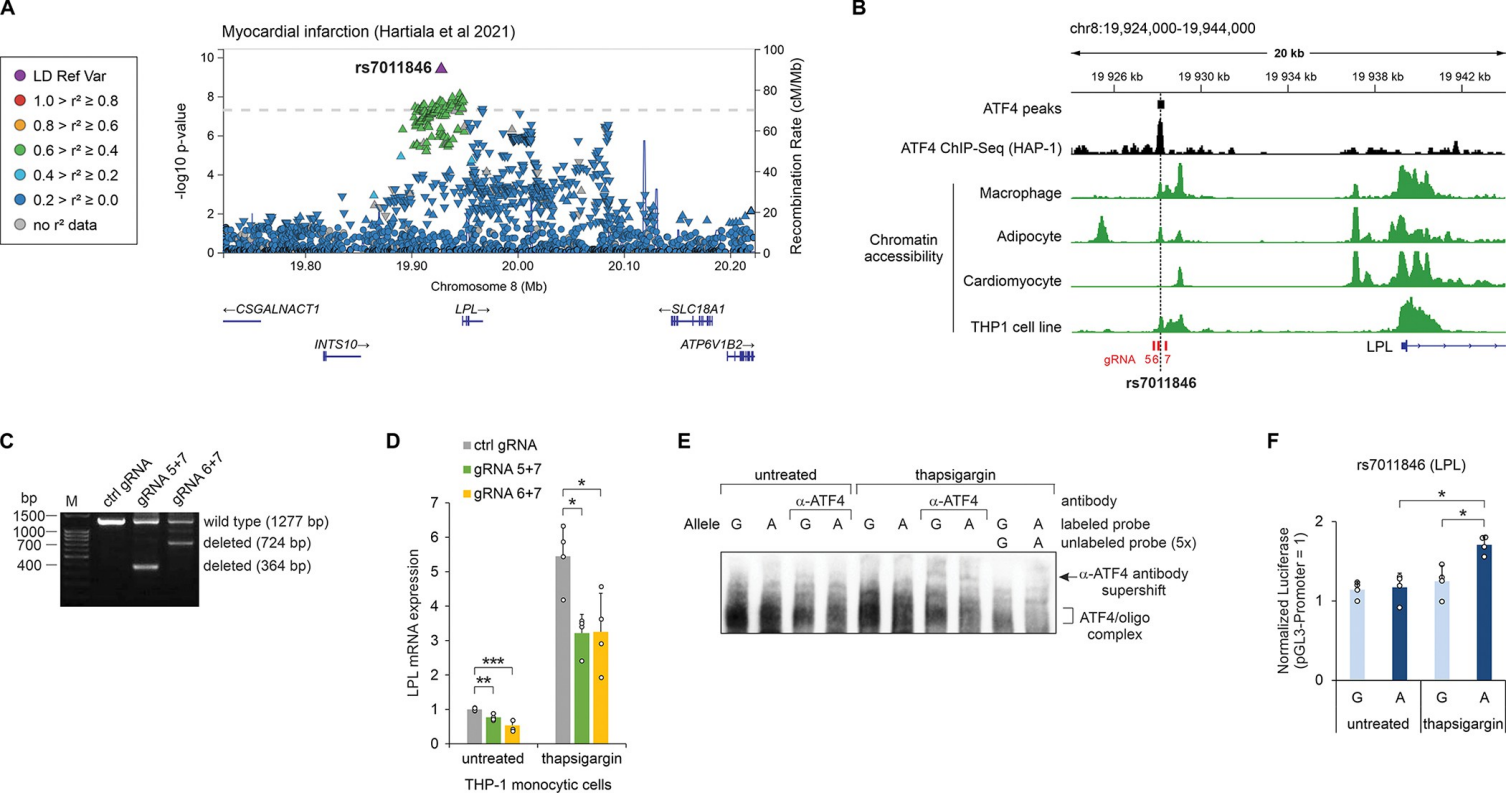
Effect of the rs7011846-harboring regulatory region on *LPL* expression in THP-1 cells. **(A)** LocusZoom plot of the myocardial infarction GWAS study conducted by Hartiala et al [55]. The trait association *p* values for all variants studied in this region are shown and the orientation of the arrowheads indicates the effect direction (GWAS Catalog accession: GCST011364). Linkage disequilibrium (LD) in the European population is shown relative to the lead SNP (rs7011846), provided by LocusZoom [98]. **(B)** Chromatin accessibility and ATF4 ChIP-Seq coverage near rs7011846. The SNP location is indicated with a vertical dashed line, and the locations of CRISPR gRNAs are shown in red. Chromatin accessibility tracks were obtained from the human single-nucleus ATAC-Seq atlas [56], except THP-1, which was obtained from GEO accession GSE96800 [100]. **(C)** PCR-based validation of CRISPR-Cas9 mediated deletion of the rs7011846 region using gRNA 5+7 or gRNA 6+7. **(D)** RT-qPCR quantification of *LPL* mRNA expression in untreated and thapsigargin-treated cells transfected with control gRNA (ctrl gRNA) or gRNAs designed to disrupt the regulatory region overlapping rs7011846 (gRNA 5+7 or gRNA 6+7). Data are presented relative to the mRNA expression level in untreated cells transfected with ctrl gRNA. **(E)** EMSA analysis for SNP rs7011846 allele-specific binding of ATF4. Biotin-labeled probes containing rs7011846 G or A alleles were incubated with nuclear extracts from untreated and thapsigargin-treated THP-1 cells. In lanes labeled α-ATF4, antibody targeting ATF4 was included in the reaction. Where indicated, unlabeled competitor probe was added to the reaction in 5-fold excess. **(F)** Luciferase reporter activity for genomic regions containing SNP rs7011846 (G/A) in untreated and thapsigargin-treated THP-1 cells. In panels D and F the mean ± SD is shown from 4–6 transfection experiments performed on separate days. *p < 0.05, **p < 0.005, ***p < 0.0005 by two-tailed *t* test with Bonferroni-Holm correction. The coordinates shown are for GRCh38.

The intergenic SNP rs7011846 is associated to MI (Fig 4A, 6A) and resides approximately 26 kb upstream from the first exon of *LPL* gene [55]. *LPL* expression in macrophages, adipocytes and cardiomyocytes has been proposed to affect cardiovascular disease, with a pro-atherogenic effect when expressed in macrophages, anti-atherogenic in adipocytes, and myopathy-promoting in cardiomyocytes [59–61]. Analysis of chromatin structure in the rs7011846 region in these cell types revealed accessible chromatin in macrophages and adipocytes, but not in cardiomyocytes (Fig 6B). Chromatin accessibility was also confirmed in the THP-1 monocytic cell line, and ATF4 binding to the chromatin region was observed in HAP-1 cells ChIP-Seq (Fig 6B). Therefore, we used THP-1 cells to study whether the rs7011846 locus is involved in the regulation of *LPL* expression. THP-1 cells were edited with CRISPR-Cas9 to generate deletions with the size of 913 bp and 553 bp, encompassing the rs7011846 (Fig 6B, 6C). The relatively large deletions were required to avoid targeting DNA repeat sequences near the SNP. The edited cells were exposed to thapsigargin, an inhibitor of ER Ca^2+^-ATP-ase, which upregulates the ATF4 ISR pathway, or left untreated. RT-qPCR showed that thapsigargin induces the expression of *LPL*, and deletion of the rs7011846 region significantly downregulates the expression of *LPL* in untreated as well as thapsigargin treated cells (Fig 6D). EMSA from THP-1 cells exposed to thapsigargin revealed that both alleles of rs7011846 form oligonucleotide–protein complexes which are supershifted by anti-ATF4 antibody (Fig 6E), confirming that endogenous ATF4 can bind to the sequences. Luciferase reporter assay demonstrated that the rs7011846 alternative allele (A) significantly upregulated the reporter expression in THP-1 cells exposed to thapsigargin while the reference allele (G) did not, indicating that the alternative allele has higher ability to activate transcription in response to stress, compared to the reference allele (Fig 6F).

## Discussion

Genetic variants in transcription factor binding sites can have a significant impact on gene expression, and understanding these effects is crucial for unraveling the genetic basis of disease. In this manuscript, we performed the genome-wide characterization of GWAS trait-linked SNPs located in the ATF4 binding sites. ATF4 has been implicated in various biological processes and diseases, including cellular stress response, development, cancer and metabolic diseases [2,3,62–65], and similarly, we found that the SNPs which reside in the chromatin binding sites of ATF4 are associated to a broad range of traits. The ability of ATF4 to carry out multiple functional roles might be, at least in part, caused by the property of ATF4 to dimerize and regulate gene expression in complex with other transcription factors [2,11]. Notably though, ATF4 ChIP-Seq data suggests that by far the most frequently bound motif for ATF4 is the C/EBP-ATF composite sequence, restricting the possible dimers. In the current paper, using ChIP-Seq data of more than 200 chromatin binding factors in HepG2 cells revealed the highest binding profile similarity of ATF4 towards several C/EBP and AP-1 family members, bZIP transcription factors that are known dimerization partners of ATF4 [11]. Interestingly, the chromatin binding profile of ATF4 also exhibits remarkable similarity to that of zinc finger protein ZNF219 and nucleosome remodeling and deacetylase complex subunit GATAD2A, but neither of the proteins has been reported to interact with ATF4 and the DNA sequence recognized by ZNF219 differs from that of ATF4 [66]. Thus, these factors could be binding to co-occurring motifs, rather than directly dimerizing with ATF4.

Intersecting our compendium of ATF4 binding sites with GWAS and PheWAS data identified 581 ATF4 motif-altering SNPs which are associated to at least one human trait. Analyzing these genetic variants in an allelic MPRA showed that in nearly 90% of cases the alleles demonstrate transcriptional activity directionally in agreement with that predicted from the motif sequence. This indicates that the ATF4 motif is accurately defined from ChIP-Seq experiments and suggests that ATF4 overwhelmingly functions as a transcriptional activator, in line functional genomics experiments in *Atf4*-deficient mouse cells [32], while an early report suggested it may also function as a repressor [67]. Importantly, luciferase assays, representing a different reporter system, confirmed several of our MPRA allelic results, and EMSA supershift assays extended the results by confirming allelic differences in ATF4 binding.

Among the SNPs for which we confirmed an allelic effect on ATF4 binding and ATF4-mediated transcriptional activity, was rs532446 which affects the expression of *GADD45A* based on eQTL data [49]. In GWAS, rs532446 is strongly associated to platelet volume, corpuscular hemoglobin and corpuscular volume, which are characteristics of blood cells belonging to the megakaryocyte and erythroid lineages. These two lineages are closely related and originate from a common progenitor [68], suggesting that GADD45A may play a role in the differentiation of the megakaryocyte-erythroid progenitor. Terminal megakaryocyte differentiation involves megakaryocyte apoptosis, required for protoplatelet formation and platelet release, and the expression of *GADD45A* was shown to be upregulated during megakaryocyte apoptosis [69]. Additionally, previous studies in mice have demonstrated that *Gadd45a* regulates hematopoietic stem cell stress response and aged *Gadd45a*^-/-^ mice had a greater number of erythrocytes in peripheral blood compared to aged *Gadd45a*^+/+^ mice [70]. As ATF4 has been implicated both in megakaryocytopoiesis [5] and erythropoiesis [71], *GADD45A* could serve as a downstream transcriptional target in these processes.

The SNP rs7011846, located approximately 26 kb upstream of *LPL* and identified as a lead SNP for myocardial infarction [55] is one of relatively few SNPs in our study which changes one ATF4 binding motif to another, rather than creating or disrupting a binding motif. Although the ability of ATF4 to bind to the CREB-C/EBP motif (rs7011846 reference allele) and the C/EBP-ATF motif (rs7011846 alternate allele) is similar based on EMSA, other factors binding to the site along with ATF4 (or besides ATF4) might be different, leading to differences between the alleles in the regulation of transcriptional activation (as seen in the reporter assay results). For example, while both the CREB-C/EBP site and the C/EBP-ATF site can be bound by ATF4-C/EBP heterodimer, the CREB-C/EBP site can also be bound by the ATF2-C/EBPα heterodimer and the homodimers of ATF2, C/EBPα and CREB [36,72]. Given the large number bZIP transcription factors capable of heterodimerization [11], other combinations of bZIP transcription factors interacting with these motifs may exist as well. Based on eQTL data, the SNP rs7011846 affects the expression of *LPL*, which is involved in the development of atherosclerosis, as enhanced expression of *Lpl* in macrophages accelerates the generation of foam cells and animals with *Lpl*^-/-^ macrophages have reduced atherosclerosis [59,73–75]. ER stress signaling and specifically the PERK-ATF4 pathway is known to be activated in macrophage foam cells due to elevated lipid levels, oxidative stress and/or calcium imbalance, and is thought to play a role in macrophage apoptosis, plaque necrosis and the clinical progression of atherosclerosis [76].

Among the SNPs selected for downstream validation experiments, the SNPs rs281785 (associated to schizophrenia and other psychiatric disorders) and rs6491544 (associated to educational attainment) revealed particularly strong allelic differences in the inducibility in response to tunicamycin and in the ability to interact with ATF4 and activate ATF4-mediated transcription. The SNP rs281785 resides approximately 33 kb upstream of TSS of *FTCDNL1* and recently a causal relationship between increased schizophrenia risk and reduced *FTCDNL1* expression was reported [77]. Previous studies have implicated ATF4 in various aspects of central nervous system functioning. For instance, *ATF4* expression is correlated with general cognitive function in a gene-based association study [78] and *ATF4* expression level was found to be markedly reduced in the frontal cortex of human patients with schizophrenia [79]. Moreover, hippocampal *ATF4* downregulation leads to defects in synaptic plasticity and memory [8] and increases neuronal excitability by reducing GABA(B) receptor level, the deficit of which has been linked to a variety of neurological and psychiatric conditions, including schizophrenia [80]. In addition, ATF4 has been shown to interact with Disrupted-in-Schizophrenia 1, a risk factor for major mental illnesses and regulate dopaminergic signaling by repressing transcription of phosphodiesterase 4D, a gene involved in psychiatric disorders [81].

A limitation of our MPRA study is that the candidate sequences were all tested using an identical minimal promoter as part of the mammalian MPRA vector [42]. Recent studies have profiled the combinatorial compatibilities of mammalian enhancers and promoters, revealing that while compatibility is often broad and the intrinsic enhancer/promoter activities determine most of the combination outcome, cases of restricted compatibility also exist [82,83]. Secondly, the MPRA experiment was only carried out in one cell line, with a small set of variants studied in follow-up experiments in other cell types. As ATF4 is expressed in many cell types across the body, it could be worth repeating the MPRA experiment in more contexts. Similarly, the principal ATF4 binding motif appeared highly similar across the current ChIP-Seq samples, but many cell lineages remain unexplored.

In summary, our application of a high-throughput reporter assay and downstream validation experiments provides new understanding of how nucleotide variation within ATF4 binding sites affects the transcriptional regulatory output of these genomic sequences. More studies, in targeted cellular and tissue contexts, will be needed to understand precisely how the altered regulatory activity leads to emergence of a trait or disease.

## Materials and methods

### Processing of ATF4 ChIP-Seq read data

The raw sequence data for human ATF4 ChIP-Seq experiments (and the corresponding input control sequencing data) was obtained from the GEO and ENCODE repositories using the accession codes listed in (S1 Table) [18–24]. The sequence reads were processed using the nf-core ChIP-Seq pipeline version 1.2.1 [84] with the GRCh38 genome and the narrow peak mode. The pipeline uses BWA for read alignment and MACS2 for peak calling. For each library, as a quality control of the ATF4-specificity of the immunoprecipitation, the enrichment of the C/EBP–ATF motif (specifically, the motif ‘MEF-Atf4-ChIP-Seq/GSE35681’ from the HOMER motif database) was quantified with HOMER (version 4.10) [85] using the MACS2-called peak summit coordinate ± 100 bp as the search region. The merged peak set across libraries was created using nf-core ChIP-Seq pipeline default settings (i.e., peaks present in at least one replicate are included and any overlap length is accepted). Peak annotation and enrichment of known and *de novo* motifs were done with HOMER (version 4.10) [85]. Gene Ontology (GO) Biological Process enrichment analysis was carried out using the g:Profiler web tool [86] (access date: 2022-11-24). GO terms with enrichment p_adj_ < 0.05 were retained, and GOSemSim [87] (version 2.8.0) was used to identify redundant GO terms (similarity measure “Rel”, cutoff 0.7) and the most significant term by p value was retained from each group of similar terms. Activity-by-contact (ABC) model predictions of regulatory element-gene connections were obtained from https://www.engreitzlab.org/resources (accessed 2023-09-07) [30]. Connections with ABC score ≥0.015 were retained and their regulatory element coordinates were intersected with ATF4 peaks of the respective cell type, requiring a minimum overlap of 50 bp.

### Cell culture and viability

HepG2 cells were cultured in Dulbecco’s Modified Eagle Medium (DMEM) (4.5 g/l D-glucose; Gibco) supplemented with 10% fetal bovine serum (FBS) and 1× penicillin-streptomycin (Pen/Strep). ATF4-293 cells (HEK293-derived cells with a tetracycline (tet)-inducible human ATF4 expression) were cultured as described previously [88]. THP-1 and K562 cells were maintained in RPMI-1640 medium (Gibco) supplemented with 10% FBS and 1× Pen/Strep. HepG2 cell viability was measured in 96-well plates by Alamar Blue (resazurin; Acros Organics) metabolic assay as described previously [21].

### Gene expression profiling datasets for defining ATF4-sensitive genes

Processed data was collected from published genome-wide gene expression profiling experiments with ATF4 activity modulation [18,21,31–34] (experiments summarized in S8 Table). Gene expression data of ATF4-293 cells [88] with and without tet-activated ATF4 expression was generated as described previously [89]. GO enrichment analysis of gene sets was carried out as described above for ChIP-Seq. The raw data from the ATF4-293 gene expression experiment is available at the ArrayExpress repository (accession number E-MTAB-3318).

### Human genetic variants in ATF4 binding regions

All NCBI dbSNP build 151 (GRCh38) [90] human common genetic variants (MAF > 0.01 in at least one dbSNP reference population; denoted by the VCF flag *COMMON*) were considered, totaling approximately 37 million variants. First, the variants were intersected with the ATF4 merged peak set (described above), resulting in 402,380 variants with overlaps to ATF4 binding regions. For these dbSNP variant rsID-s, the alleles and flanking genomic sequences were obtained using Ensembl BioMart (Ensembl Variation 104, Human short variants GRCh38; www.ensembl.org/biomart/martview) and checked whether the allelic sequences alter (overlap) an ATF4 binding motif. Specifically, whether for at least one of the alleles, the variant sequence surrounded by 9 bp genomic flanks generates a match to a 10-mer motif. The following motifs were checked from both DNA strands: NTGATGNAAN (C/EBP–ATF), NTGACGTCAN (CRE), NTGACGTGNC (BATF–ATF) and NTGACGNAAN (CREB–C/EBP). As a comparison, position weight matrix (PWM) scoring of the allelic sequences was carried out with HOMER (version 4.10) [85] and the motif match threshold selected automatically by the HOMER motif finding algorithm was used.

To check the ATF4 peak- and motif-overlapping variant sets for enrichment of eQTL variants, the CAVIAR finemapping results for GTEx v8, along with genomic annotations of regulatory features for each GTEx study variant, were downloaded from https://gtexportal.org/home/datasets (accessed 2023-09-02) [38]. All GTEx tissues were included and all variants with eQTL posterior inclusion probability (PIP) >0.2 were retained. For testing overlap enrichment, the overall pool of genetic variants was the intersection of all variants considered in our study (described above) and all variants considered in the GTEx eQTL pipeline (MAF ≥ 1% in GTEx [38]), a total of 10,364,550 shared variants.

The Open Targets Genetics Portal (June 2021 data release) [39,40], which aggregates and processes genetic association data from the GWAS Catalog and other sources, was used to link genetic variants to phenotypes as well as to link variants to potential target genes. Each motif-overlapping genetic variant (described above) was used in an Open Targets GraphQL API query to identify whether it has been associated, either as an index SNP (lead SNP) or a proxy SNP (tag SNP), to any human trait. Proxy SNP-s based on linkage disequilibrium (LD) were used as precalculated by the Open Targets Genetics Portal, which uses for each GWAS study the most closely matching 1000 Genomes Phase 3 superpopulation as the LD expansion haplotype reference panel [39]. When available, posterior probabilities from credible set analysis were also obtained from Open Targets. A second Open Targets GraphQL API query was used to evaluate likely target genes for each variant based on the Open Targets Variant-to-Gene (V2G) pipeline, which integrates multiple lines of evidence, such as QTL and chromatin interaction data as well as distance to TSS, in a weighted scoring schema [39].

### Sequence design and plasmid library construction for MPRA

To construct the MPRA plasmid library, 230 nt single-stranded DNA oligonucleotides were designed in the format 5′-ACTGGCCGCTTCACTG-(175 nt query sequence)-GGTACCTCTAGA-(10 nt barcode)-AGATCGGAAGAGCGTCG-3′ and ordered as pooled synthesis of 7500 oligonucleotides from Agilent Technologies. In total, 581 genetic variants were selected for the MPRA. The genetic variant was placed at the center of the 175 nt query sequence region, surrounded by flanking sequence from the GRCh38 human reference genome. For candidate SNP-s located in a promoter region (defined as −1000…+1000 bp relative to a TSS in GENCODE transcriptome release 32), the query region was cloned to the plasmid in the orientation matching the strand of the nearest TSS. For non-promoter regions, the positive strand of the GRCh38 genome was cloned. To allow for restriction enzyme-based cloning, any *Kpn*I (GGTACC), *Xba*I (TCTAGA) and *Sfi*I (GGCCNNNNNGGCC) sites in the genome sequence were removed by a 1 bp substitution at the base furthest from the SNP of study (all edit locations are noted in S13 Table).

As positive control sequences, 14 human genome sequences harboring known functional ATF4 response elements were included in the library. These sequences were additionally used to design a set of negative controls by introducing point mutations aimed to disrupt the known ATF4 binding site. Additionally, 5 viral promoter or enhancer fragments and 22 artificial multimers of transcription factor motifs were included as controls. Scramble controls were included for a randomly selected ~20% subset of candidate SNP regions (121 regions out of 581). All oligonucleotides selected for study are detailed in S13 Table.

The DNA barcode sequences associated to MPRA query sequences (and later incorporated in the reporter mRNA transcript as the assay read-out) were selected out of a pool of all possible 10-mers, filtered to exclude barcodes with a >3 bp long homopolymer tract, GC content >60% or <30%, a restriction site for *Xba*I or *Kpn*I, a poly-adenylation consensus sequence (AATAAA), a match to any transcription factor binding motif in the HOMER motif database (as scored by HOMER, including 4 bp flanking sequence from the vector backbone) or an exact match to the seed sequence (positions 2–8) of any human mature microRNA (miRBase version 22). Finally, the DNABarcodes R package (version 1.12) [91] was used to exclude sequences with self-complementarity and to filter the barcode pool to have a minimum sequence difference (hamming distance) of 3 bp between any two barcodes. Barcodes from the final barcode pool were assigned to MPRA query sequences at random without reuse. For candidate SNP-s, each allele was synthesized with 6 different barcodes, while positive and negative control sequences were synthesized with 3 different barcodes each (S13 Table).

The plasmid library preparation followed the procedure described by Melnikov et al [42]. The oligonucleotide library was amplified by emulsion PCR using primers MPRA-Sfi-F (5′-GCTAAGGGCCTAACTGGCCGCTTCACTG-3′) and MPRA-Sfi-R (5′-GTTTAAGGCCTCCGTGGCCGACGCTCTTCCGATCT-3′), the Phusion Hot Start II High-Fidelity DNA Polymerase (Thermo Scientific) and, to prepare the emulsion, the Micellula DNA Emulsion & Purification Kit (EURx, # E3600-01). The emulsion PCR products were size-selected and purified by agarose gel electrophoresis, digested by *Sfi*I, and ligated into the 2.5-kb backbone of *Sfi*I-cut plasmid pMPRA1 (Addgene #49349) [42]. *E*. *coli* XL10-Gold Ultracompetent Cells (Agilent Technologies) were transformed with ligation mixture and transformants were grown overnight in LB medium containing 100 μg/ml ampicillin. To maintain library complexity, transformation yield was monitored to be at least 100 colony forming units per library oligonucleotide. From this culture, the intermediate step library plasmids were isolated, digested by *Kpn*I and *Xba*I, and the 1.8-kb *Kpn*I/*Xba*I fragment of plasmid pMPRAdonor2 (Addgene #49353) [42] was ligated into the *Kpn*I/*Xba*I-cut intermediate library plasmids to insert the minimal promoter and *luc2* ORF between the MPRA query sequence and the MPRA barcode. *E*. *coli* XL10-Gold Ultracompetent Cells (Agilent Technologies) were transformed with the ligation mixture and grown as above to obtain the final MPRA library plasmid pool.

### MPRA cell culture experiment and sequencing

6×10^6^ HepG2 cells per replicate were transfected with 22 μg MPRA plasmid library using the Lipofectamine 3000 transfection reagent and Opti-MEM I reduced-serum medium (both Thermo Scientific). 9 h after transfection, the medium was replaced with regular growth medium (described above) with or without 2 μg/ml tunicamycin, and the cells were harvested 17 h later. To generate replicates, the transfection and treatment of cells were repeated on separate days (n = 5).

Total RNA was extracted from the cells using the RNeasy Midi Kit (Qiagen) according to the procedure described by the manufacturer (including the on-column DNase treatment), and poly(A)+ RNA was subsequently isolated using Dynabeads Oligo(dT)_25_ beads (Invitrogen). Poly(A)+ RNA was treated with DNase I (Thermo Scientific) and purified using the RNeasy Micro kit (Qiagen).

Poly(A)+ RNA (5 μg) was reverse-transcribed with SuperScript III Reverse Transcriptase (Invitrogen) using primer #1073 (5′-AATGATACGGCGACCACCGAGATCTACACNNNNNNNNNNNNACACTCTTTCCCTACACGACGCTCTTCCGA-3′), which contains a 12-nt UMI (unique molecular identifier) and the Illumina P5 adapter sequence. The resulting single-stranded cDNA was treated with RNase A (Thermo Scientific) and purified with AMPure XP beads (Beckman, A63882) using 1.8× beads to sample ratio. To generate the MPRA plasmid library ‘input’ samples for sequencing, we performed the synthesis of DNA with Phusion Hot Start II High-Fidelity DNA Polymerase using the primer #1073 (described above) and the plasmid library as template, and the products were purified with QIAquick PCR Purification Kit (Qiagen).

To prepare the NGS libraries, the cDNAs and the input samples were amplified for 14 cycles with Phusion Hot Start II High-Fidelity DNA Polymerase, using primers #1080 (5′-GTGACTGGAGTTCAGACGTGTGCTCTTCCGATCTCGAGGTGCCTAAAGGACTGAC-3′) and #1072 (5′-AATGATACGGCGACCACCGAGA-3′). The PCR products were cleaned up with QIAquick PCR Purification Kit and subsequently used as template in an indexing PCR reaction of 6 cycles using primer #1072 (as above) and an i7 index primer (7001–7014 from the Lexogen 6-nt i7 index plate). The PCR products were size selected by agarose gel electrophoresis and extracted with QIAquick Gel Extraction Kit, followed by purification with AMPure XP beads (beads to sample ratio 1:1.8).

The Illumina-indexed MPRA libraries were combined in equimolar ratio and single-end sequenced on an Illumina NextSeq 500 instrument using the following program: Read 1: 100 bp, Index 1: 6 bp (i7 index), Index 2: 12 bp (UMI in place of the i5 index). The mean sequencing depth was 6.8×10^6^ reads per library (range 5.2×10^6^ to 9.3×10^6^). The raw sequencing data is available at NCBI GEO under the accession GSE225216.

### MPRA sequencing data processing

Reads were trimmed to retain 35 bp from the start of the read (expected to contain the 10-bp MPRA sequence barcode followed by 25 bp of constant flanking sequence). The reads were then mapped using Bowtie (version 1.3.0) [92] to a synthetic genome consisting of 35 bp contigs (each of the possible 10-bp barcodes attached to 25 bp of constant sequence). In mapping, one nucleotide mismatch was allowed and any multimapping reads were discarded. On average, 95.6% of reads per library (range 94.8% to 95.9%) were mapping, i.e., corresponding to an expected MPRA barcode. After mapping, UMI deduplication was carried out using UMI-tools (version 1.1.0) [93] to filter out likely PCR duplicate reads using default settings. On average, 83.3% (79.1–86.4%) of RNA library reads and 96.5% (96.3–96.7%) of input plasmid DNA library reads were retained after UMI deduplication. Barcodes were filtered to retain those with ≥5 reads in every input DNA plasmid library replicate and, for at least one cell treatment condition, ≥5 reads in every RNA library replicate, resulting in a count matrix with 6983 barcodes (93.1%) representing 1334 MPRA query sequences (99.7%). Barcode counts in each library were then normalized for sequencing depth (counts per million). For each barcode, abundance in RNA libraries was normalized to the abundance of the barcode in the input plasmid DNA pool, and further normalized to the median level of scramble controls within each RNA library. Finally, to obtain the normalized reporter activity for each MPRA query sequence, barcodes corresponding to identical query sequence were averaged. To determine differential reporter activity in response to tunicamycin treatment, the normalized reporter activities were voom-transformed and analyzed with limma (version 3.38.3) [94]. Statistical testing of allelic effects was carried out using MPRAnalyze (version 1.8.0) [45] with the barcode-level counts (after UMI deduplication) as input. MPRAnalyze models noise at the barcode level in both DNA and RNA to minimize barcode-specific effects and uses a likelihood ratio test for allelic effects [45]. FDR was used to correct for multiple testing, and FDR < 0.05 was considered significant.

### Luciferase reporter constructs and dual-luciferase reporter assay

PCR was used to amplify 175-bp (or 163-bp in the case of SNP rs6491544) human genomic DNA fragments centered on the reference (ref) or the alternative (alt) allele of the following SNPs (the alt and ref nucleotides, and PCR primers used, are indicated in parenthesis): rs532446 (ref C; alt T; primers #1113 (5′-CTCGGTACCTGTGGTAGGTGGGGGTCA-3′) and #1114 (5′-CACACGCGTGGGCTGACTGCTGACTCA-3′)), rs2718215 (ref T; alt C; primers #1131 (5′-CTCGGTACCTGCCATTTTAACTATTGTTAAGTGT-3′) and #1132 (5′-CTCACGCGTTACCAGGCATGGGATGT-3′)), rs6491544 (ref T; alt C; primers #1115 (5′-CTCGGTACCTATTTGAAAGAAAAGCTGTGCTTCC-3′) and #1116 (5′-CCCACGCGTTTACTTCATCTTGCTTCATCTCT-3′)), rs281785 (ref A; alt G; primers #1119 (5′-CTCGGTACCACCCAAGTGGATATGTCAGTT-3′) and #1120 (5′-CTCACGCGTGCCCAAAGCACTGGGATTAC-3′)), and rs7011846 (ref G; alt A; primers #1143 (5′-CTCGGTACCGGAGACTGGGGTAGGAGG-3′) and #1144 (5′-CACACGCGTCTTTTCCTTCCTTCCTTTCTTTTTT-3′)). The 230-nt oligonucleotide library (Agilent Technologies) was used as the PCR template. *Acc65*I and *Mlu*I sites were introduced by PCR at the ends of genomic DNA fragments and the PCR products were inserted into *Acc65*I/*Mlu*I-digested plasmid pGL3-Basic (Promega), which encodes firefly luciferase. In the case of rs7011846, the *Acc65*I/*Mlu*I-digested PCR products were also inserted into *Acc65*I/*Mlu*I-digested plasmid pGL3-Promoter (Promega). The plasmids were verified by Sanger sequencing.

HepG2 cells were grown in 96-well plates and co-transfected with 60 ng of firefly luciferase plasmid (either pGL3-Basic or the pGL3-Basic-derived reporter constructs with allelic sequences, or, for optimization experiments, the MPRA library) and 9 ng of *Renilla* luciferase plasmid (pRL-TK; Promega) using polyethylenimine (PEI-MAX 40,000; Polysciences Inc. #24765). Where indicated, 10 ng of expression plasmid encoding human ATF4 (ATF4-pCG) [95], or the corresponding empty vector (pCG), was included in the transfection mixture. K562 or THP-1 cells were co-transfected with pRL-TK and either with pGL3-Basic-derived reporter constructs (K562) or pGL3-Promoter-derived reporter constructs (THP-1) using the Invitrogen Neon Transfection System (10 μl kit) at settings 1000 V/50 ms/1 pulse for K562 and 1100 V/30 ms/1 pulse for THP-1 cells. Transfections were done in duplicate wells, which were averaged prior to statistical analysis. For statistical analysis, transfections done on separate days were considered as separate experiments (n = 4–5).

Transfected cells were cultured for 18 h, after which the cells were either mock-treated or treated with medium containing 2.5 μg/ml tunicamycin for 12 h (HepG2), 20 μM sodium arsenite (Sigma) for 8 h (K562) and 1 μM thapsigargin (Calbiochem) for 12 h (THP-1). In the luciferase experiment with the MPRA library, cells were exposed to 2 μg/ml tunicamycin for the indicated time. Firefly and *Renilla* luciferases were measured using a dual-luciferase assay (Promega) as described previously [96]. In each sample, firefly luciferase activity was normalized to *Renilla* luciferase activity, and results are presented relative to the activity of the empty vector (pGL3-Basic) in the same experimental conditions.

### Electrophoretic mobility shift assay (EMSA)

For each allele, the forward and reverse strand were synthesized as complementary 31-nt 5′-biotin-labeled DNA oligonucleotides (Microsynth): 5′-GTGCTAGTTTCATCA[C/T]CAGGATTTTCTGTGG-3′ for rs532446; 5′-AAATGCTTGTATGAT[A/G]CAACTCTACCTAAAT-3′ for rs281785; 5′-TCACTATTTCTACAA[T/C]TTCATCACCCCACAC-3′ for rs2718215; 5′-CTCTGTTACAATGCA[T/C]GATGAAATACCGTGT-3′ for rs6491544 and 5′-GTGAGCCATGATTGC[G/A]TCACTGCACTCCATC-3′ for rs7011846. Unlabeled identical sequences were synthesized for competition assays. The single-stranded complementary oligonucleotides were annealed to obtain double-stranded probes for EMSA.

HepG2 cells were transfected with plasmid ATF4-pCG using the Lipofectamine 3000 transfection reagent as described above for the MPRA library and harvested 24 h post-transfection. ATF4-293 cells were treated for 24 h with 1 μg/ml tet in regular medium to induce ATF4 expression. To induce cellular stress response, K562 and THP-1 cells were cultured with sodium arsenite (0 and 20 μM) for 6 h or with thapsigargin (0 and 1 μM) for 10 h, respectively.

Nuclear proteins were extracted using the NE-PER Nuclear and Cytoplasmic Extraction Reagents (Thermo Scientific) and EMSA was carried out using the LightShift Chemiluminescent EMSA kit (Thermo Scientific) according to the manufacturer’s instructions. Briefly, labeled probes were bound to 5 μg nuclear protein extract pre-incubated with poly-dI-dC in 1x binding buffer. For competition assay, a 5-fold excess of unlabeled probe was included in the EMSA reaction. For supershift assays, 1.2 μg of anti-ATF4 antibody (sc-200; Santa Cruz Biotechnology) was used. Completed reactions were run on 5.5% native polyacrylamide gel and transferred to a Hybond-N+ membrane (Amersham) using a Trans-Blot SD semi-dry transfer cell (BioRad). DNA was crosslinked to membrane in a UV Stratalinker 1800 (Stratagene). Blots were assayed by chemiluminescence and imaged on ChemiDoc XRS+ (BioRad).

### Western blotting

Immunoblotting was carried out as described previously [21]. Briefly, nuclear extracts were prepared from ATF-293 cells grown with or without tetracycline, as described in EMSA section. Protein concentration in the nuclear lysates was determined using the BCA protein assay (Pierce), and an equal amount of protein (20 μg) was loaded per lane. The blots were incubated with rabbit anti-ATF4 polyclonal antibody (1:5000 dilution; Santa Cruz Biotechnology sc-200), followed by incubation with horseradish peroxidase-conjugated goat anti-rabbit IgG secondary antibody (1:3000 dilution; Cell Signaling Technology #7074). Blots were treated with Immobilon chemiluminescent reagent (EMD Millipore) and proteins were visualized with ChemiDoc XRS+ detection system (BioRad).

### CRISPR-Cas9-mediated disruption of SNP loci in cell culture

Mutations in the regions containing SNP rs532446 or rs7011846 were generated by Alt-R CRISPR-Cas9 System (crRNA-tracrRNA duplexes and S. p. Cas9 nuclease; Integrated DNA Technologies (IDT)). The solutions of crRNA-tracrRNA duplexes (gRNAs) were prepared by mixing target-specific crRNAs with the universal tracrRNA at equimolar concentration in Duplex Buffer (IDT), incubating the mixture at 95°C for 5 min, at 78°C for 10 min, and then cooling the mixture to 25°C. The solutions of ribonucleoprotein (RNP) complexes were made by mixing Cas9 enzyme with gRNAs at equimolar ratio and incubating at room temperature for 30 min. The RNP complexes were introduced into the cells by electroporation using the Neon Transfection System (Invitrogen).

To disrupt the rs532446-overlapping C/EBP-ATF motif by introducing short indels, K562 cells (which are heterozygous for rs532446) were transfected with an equimolar mixture of two gRNAs containing the allele-specific sequences 5′-GATGTGCTAGTTTCATCACC-3′ (gRNA 1) and 5′-GATGTGCTAGTTTCATCATC-3′ (gRNA 2), corresponding to the reference and alternative allele of rs532446, respectively. To delete a 163-bp fragment encompassing the SNP rs532446 from the K562 genome, cells were transfected with the equimolar mixture of gRNA 3 (5′-AAGTCCAGGGACACTCTAGT-3′) and gRNA 4 (5′-CTAAAGGAATTAGTCACGGG-3′). For both types of editing, 3x10^5^ K562 cells in 10 μl resuspension buffer R (IDT) were transfected with 1.6 μM Cas9-RNP at a setting 1600 V/10 ms/3 pulses and transferred into a regular medium without Pen/Strep.

To disrupt the SNP rs7011846 regulatory element, THP-1 cells were transfected with the equimolar mixture of gRNA 7 (5′-GTATTACTAGCATGAGGTAC-3′) and either gRNA 5 (5′-CAGCATGTGGAGAATGACTA-3′) or gRNA 6 (5′-TCAGCTCAGGCAATCCTACC-3′) resulting in the deletion of 913 or 553 bp encompassing the SNP rs7011846. 5x10^5^ THP-1 cells in 10 μl resuspension buffer R (IDT) were transfected with 2 μM Cas9-RNP at a setting 1050 V/30 ms/2 pulses and transferred into a regular medium without Pen/Strep.

For both cell lines, control experiments were conducted with a predesigned Alt-R CRISPR-Cas9 human HPRT positive control crRNA (IDT) targeting the hypoxanthine phosphoribosyltransferase gene. Independent biological replicates were obtained by transfections done on different days in all the CRISPR-Cas9 experiments described above.

48 h after electroporation, the transfected K562 and THP-1 cells were subdivided for treatments and seeded into a fresh regular medium. After overnight growth, K562 cells were treated with 20 μM sodium arsenite (Sigma) for 9 h or left untreated, and THP-1 cells were treated with 1 μM thapsigargin (Calbiochem) for 12 h or left untreated. Total RNA was extracted from cells using TRIzol (Invitrogen) and quantified with the NanoDrop 1000 spectrophotometer (Thermo Scientific). RNA samples were treated with DNase I and used for cDNA synthesis with FIREScript reverse transcriptase (Solis BioDyne) according to the manufacturer’s instructions. Real-time PCR was performed as described previously [97]. The primer pairs used were 5′-GTGCTGGTGACGAATCCAC-3′ and 5′-CATGTAGCGACTTTCCCGGC-3′ for human *GADD45A* mRNA amplification, 5′-AGGCACCTGCGGTATTTGTG-3′ and 5′-GATTCGCCCAGTTTCAGCC-3′ for human *LPL* mRNA amplification, and 5′-TGCACCACCAACTGCTTAGC-3′ and 5′-GGCATGGACTGTGGTCATGAG-3′ for the amplification of glyceraldehyde-3-phosphate dehydrogenase (*GAPDH*) mRNA used to normalize gene expression.

Genomic DNA was extracted from cells 48 h after transfection and the genomic region containing the CRISPR target sites in the rs7011846 or rs532446 locus was amplified with Phusion Hot Start II High-Fidelity DNA Polymerase, using primers specific for the rs7011846 locus (5′-CTGCTCATGGTACGCTGACATTAC-3′ and 5′-TGAAACTACCAGGCTCAAGCAG-3′) or for the rs532446 locus (5′-TGGTGACGGTAAGGGACTGG-3′ and 5′-TGACTCCTTAATGAGGGGTGAGC-3′). To evaluate deletion efficiency, the PCR products were resolved on 1.5% agarose gel, stained with ethidium bromide and visualized by UV light. The CRISPR-Cas9 editing efficiency of gRNAs 1 and 2, which aimed to introduce short indels, was determined by TIDE analysis of Sanger sequences [57].

## Supporting information

S1 Fig Motif logos for the most significant *de novo* motif detected in ATF4 ChIP-Seq peaks by cell type. Motif discovery was performed using HOMER and the enrichment statistics are shown beside the motif logo.
(PDF)

S2 Fig Comparison of ATF4 binding profile in bortezomib-treated and untreated HepG2 cells. **(A)** Number of overlapping and unique peaks for the treatments. **(B)** Fraction of peaks shared between treatments as a function of peak strength. Peaks were ranked by the MACS2 q-value within the library. **(C)** Motif logos and enrichment statistics for the most significant motif discovered by HOMER *de novo* motif finding using peak regions called for each treatment.
(PDF)

S3 Fig Comparison of ATF4 binding profile in HAP1 cells based on ChIP-Seq experiments carried out using three different anti-ATF4 antibodies. **(A)** Number of overlapping and unique peaks for the different antibodies. **(B)** Fraction of peaks shared between antibodies as a function of peak strength. Peaks were ranked by the MACS2 q-value within the library. **(C)** Motif logos and enrichment statistics for the most significant motif discovered by HOMER *de novo* motif finding using the peak regions called for each antibody.
(PDF)

S4 Fig Gene ontology biological process enrichment analysis for genes with ATF4 binding at the TSS/promoter or at distal regulatory elements predicted to be connected based on the activity-by-contact (ABC) model. **(A)** Genes from the 200 strongest TSS/promoter peaks per cell type. **(B)** Genes with a TSS-proximal ATF4 peak in multiple cell types. **(C)** Genes connected to the 250 strongest non-promoter peaks (enhancer-gene connections predicted by the ABC model). All four cell types with available ABC model data are shown. ABC connections with score > = 0.015 were retained, and up to top 3 genes by ABC score were considered for each peak. **(D)** Distances of the regulatory element-gene connections used in panel C. In all panels, one ChIP-Seq library was used to represent each cell type (the library with the most peaks called).
(PDF)

S5 Fig Gene ontology biological process enrichment analysis for genes with ATF4-sensitive expression in RNA profiling studies. **(A)** Genes detected in multiple studies. **(B)** Genes detected in individual studies.
(PDF)

S6 Fig Comparison of consensus sequence matching and position weight matrix (PWM) scoring for finding genetic variants that overlap a C/EBP-ATF motif within ATF4 ChIP-Seq peaks. **(A)** Motif logo and occurrence statistics of the most strongly enriched *de novo* motif identified by HOMER within ATF4 peaks in the current study. The C/EBP-ATF consensus sequence (NTGATGNAAN) and the ATF4 motif from the HOMER motif catalog are shown for comparison. **(B)** Number of genetic variants found as motif overlapping. All genetic variants falling within ATF4 ChIP-Seq peaks (merged across all ATF4 IP libraries) were considered with all variant alleles when searching for motif matches. The CREB-C/EBP consensus was defined as NTGACGNAAN. **(C)** HOMER motif match score distribution for all variants that matched the HOMER *de novo* PWM for ATF4 with any allele. The highest scoring allele for each variant was retained. The fraction of variants that matched a consensus sequence is shown.
(PDF)

S7 Fig MPRA experiment optimization and reproducibility. **(A)** Time-course of luciferase reporter activity of MPRA library (in bulk) in untreated or tunicamycin-treated HepG2 cells. The mean ± SD is shown from 2 experiments performed on separate days. **(B)** Analysis of cell viability by Alamar Blue assay in HepG2 cells untreated or treated with tunicamycin for 18 or 24 h. The mean ± SD is shown from 2 (untreated) or 3 (tunicamycin) independent experiments. **(C)** Sample correlation for MPRA barcode counts (normalized for sequencing depth). All 6985 barcodes that passed minimum count filter were used. **(D)** Sample correlation at the level of log2-transformed ‘MPRA activity’ (RNA signal normalized to input DNA signal). Pairwise Pearson correlation is shown above the diagonal.
(PDF)

S8 Fig MPRA results for control sequences included in the library. **(A)** Reported ATF4-responsive elements from human genes. For each element, the wild type (WT) sequence and an engineered mutation (MUT) was assayed. **(B)** Fragments of viral promoters or enhancers, or artificial sequences containing known transcription factor response elements. In both A and B, the bars show the mean reporter activity (RNA normalized to DNA), the error bars the SD, and the dark gray dots show results from individual experiments (n = 5 per condition).
(PDF)

S9 Fig MPRA allelic effect distribution by treatment, type of regulatory element, and type of motif disrupted. **(A)** Number of MPRA variants with a significant (MPRAnalyze FDR < 0.05) allelic effect by treatment. **(B)** Effect of treatment on allelic effect size. All MPRA variants with a significant allelic effect in at least one treatment were included and are shown ranked by treatment difference. **(C)** Allelic effect detection by genomic location (promoter or distal). **(D)** Distribution of individual allelic effect sizes, showing the regulatory element type. All 581 variants were included and shown ranked by effect size. **(E and F)** MPRA allelic effects by directional concordance with motif disruptions predicted from sequence. Significant MPRA allelic effects were categorized as motif-concordant if the higher activity allele created a known ATF4 binding motif, and motif-discordant if the higher activity allele disrupted an ATF4 motif. Motif concordance is shown stratified by genomic location (E) and type of motif disrupted (F). In panels C, E and F, the number of variants in forming each set is reported on the bar segments. Promoter regions were defined as TSS +/- 1kb. Ctrl, Control (untreated); Tun, Tunicamycin.
(PDF)

S10 Fig ATF4 DNA binding activity in nuclear extracts of ATF4-293 cells. Cells were untreated (-) or treated with tetracycline (+) to induce ATF4 overexpression from a stably integrated tetracycline-upregulated expression construct. **(A)** ATF4 protein level in nuclear extracts (20 μg per line) determined using Western blotting. **(B)** EMSA of ATF4 DNA binding ability. Biotin-labeled oligonucleotides corresponding to either the ref or the alt allele of the SNP were used as probes. In lanes labeled α-ATF4, antibody targeting ATF4 was included in the reaction. Where indicated, unlabeled competitor probe was added to the reaction in 5-fold excess.
(PDF)

S1 Table ATF4 ChIP-Seq datasets used in the study. (XLSX)

S2 Table Per-library metrics of ATF4 ChIP-Seq datasets reprocessed with an identical pipeline. (XLSX)

S3 Table ATF4 peaks called in individual libraries. Per-library peaks calls were combined into one table and sorted by coordinate. Genes with transcription start sites (TSS-s) within +/- 1 kb of the peak summit coordinate are shown (based on GENCODE version 32).
(XLSX)

S4 Table Merged peak set across all ATF4 ChIP-Seq experiments. The regions were annotated using HOMER. The true/false columns at the right of the table indicate which individual experiments contributed peaks to each merged interval.
(XLSX)

S5 Table Enrichment of known motifs (the HOMER motif collection) in each ATF4 ChIP-Seq library. The peak summit coordinates +/- 100 bp were used as the search regions in HOMER.
(XLSX)

S6 Table De novo motif analysis for each ATF4 ChIP-Seq library. The peak summit coordinates +/- 100 bp were used as the search regions in HOMER.
(XLSX)

S7 Table Activity-by-contact (ABC) model predictions of regulatory element-gene connections for ATF4 ChIP-Seq peaks. ABC connections for HepG2, K562, HAP1 and Erythroblasts were obtained from https://www.engreitzlab.org/resources (accessed 2023-09-07; Nasser et al 2021, PMID 33828297; ABC score > = 0.015) and intersected with ATF4 peaks of the respective cell type (minimum overlap 50 bp).
(XLSX)

S8 Table ATF4-sensitive (putatively ATF4-activated) genes based on gene expression profiling studies. FC, fold change; KO, knock-out; WT, wild type.
(XLSX)

S9 Table Genetic variants that are located in ATF4 binding regions and alter ATF4 binding motifs. (XLSX)

S10 Table Enrichment of SNPs overlapping ATF4 peaks/motifs for finemapped eQTLs (GTEx v8, CAVIAR, PIP>0.2, any tissue). Finemapping results and genomic annotations of regulatory features were downloaded from https://gtexportal.org/home/datasets (accessed 2023-09-02).
(XLSX)

S11 Table Trait associations for ATF4 motif-altering SNP-s. Allele frequencies are shown for the ALT allele, based on gnomAD v2.1. Open Targets was used to query associations for the variants in S9 Table. In parenthesis after each trait is the index SNP association p-value, the index SNP rsID and the LD R2 between the query SNP and the index SNP. Population abbreviations for the gnomAD v2.1 whole-genome dataset: AFR, African/African-American; AMR, Latino/Admixed American; ASJ, Ashkenazi Jewish; EAS, East Asian; FIN, Finnish; NFE, Non-Finnish European; OTH, Other (population not assigned).
(XLSX)

S12 Table Genes linked to ATF4 motif-altering SNP-s (S9 Table) based on nearest TSS-s, the Open Targets Variant-to-Gene pipeline, ATF4-sensitive genes based on literature, GTEx v8 finemapped eQTLs (CAVIAR posterior probability >0.1 in any tissue), and activity-by-contact (ABC) model (score > = 0.015 in HepG2, K562, HAP1, Erythroblast or THP-1). (XLSX)

S13 Table Details of the sequences assayed in the MPRA. Oligonucleotides corresponding to the tested genetic variants as well as the MPRA positive and negative controls are shown.
(XLSX)

S14 Table Normalized reporter activity of all measured MPRA query sequences, shown separately for each cell culture experiment. Individual barcodes were aggregated by averaging.
(XLSX)

S15 Table MPRA allelic effect analysis results. The motif alterations predicted for the variants and variants’ genomic locations (promoter/distal) are additionally shown. Promoter regions were defined as TSS +/- 1kb (GENCODE v38).
(XLSX)

S1 Data Numerical data. (XLSX)
