# Supplementary material for: Genome-wide census of ATF4 binding sites and functional profiling of trait-associated genetic variants overlapping ATF4 binding motifs
Source: PLoS Genet. 2023 Oct 31;19(10):e1011014. doi: 10.1371/journal.pgen.1011014 (PMC10637723; doi:10.1371/journal.pgen.1011014)
Supplement: S1 Fig — Motif discovery was performed using HOMER and the enrichment statistics are shown beside the motif logo. (PDF) [file pgen.1011014.s001.pdf]

Supplementary Figure S1

Cell type	HOMER top *de novo* motif	Enrichment P value	% of target sequences	% of background sequences
**All combined**	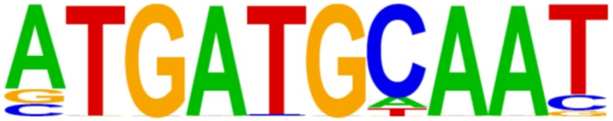	1e-208374	67.25%	1.73%
**HAP1**	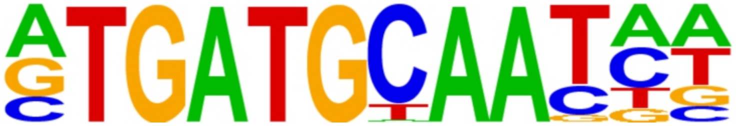	1e-39390	70.46%	1.45%
**HepG2**	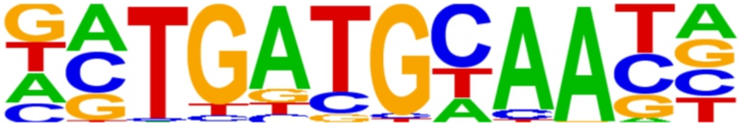	1e-10199	72.45%	2.01%
**K562**	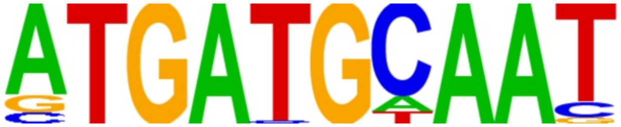	1e-42662	63.74%	1.64%
**MSC**	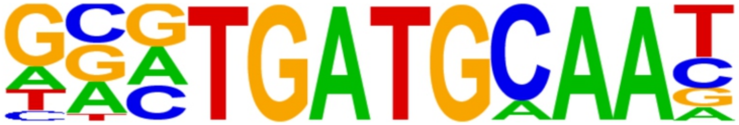	1e-578	56.57%	0.57%
**DLD1**	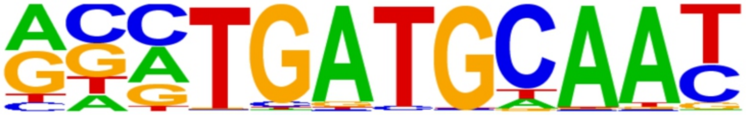	1e-395	69.00%	1.18%
**HUDEP2**	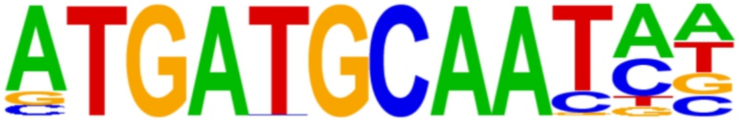	1e-6973	64.37%	0.39%
**Erythroblast**	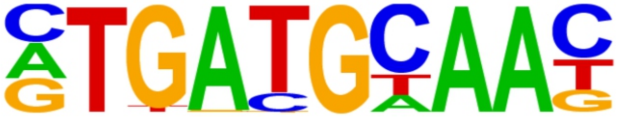	1e-4094	65.18%	1.96%
