# Supplementary figures and images for: Genome-wide census of ATF4 binding sites and functional profiling of trait-associated genetic variants overlapping ATF4 binding motifs
Source: PLoS Genet. 2023 Oct 31;19(10):e1011014. doi: 10.1371/journal.pgen.1011014 (PMC10637723; doi:10.1371/journal.pgen.1011014)

# Supplementary Figure S2

A

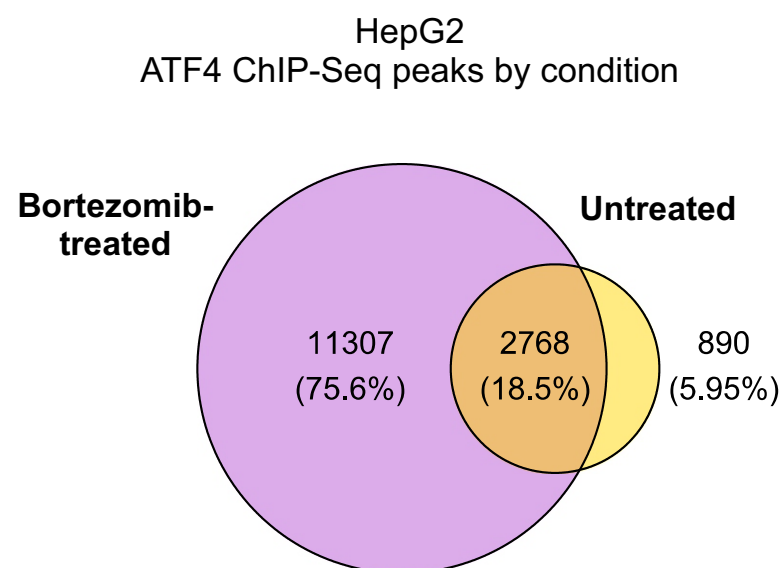

B

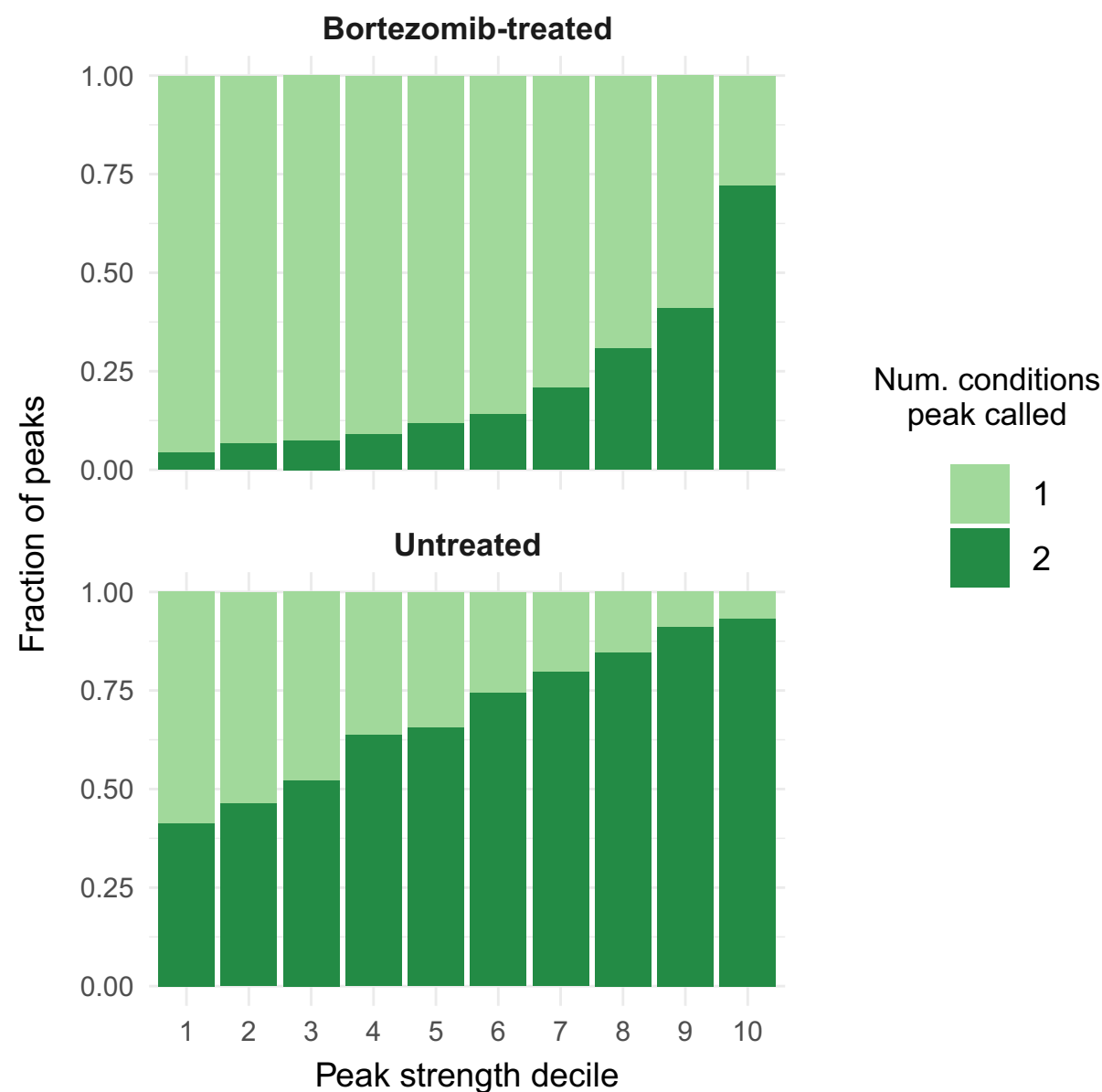

C

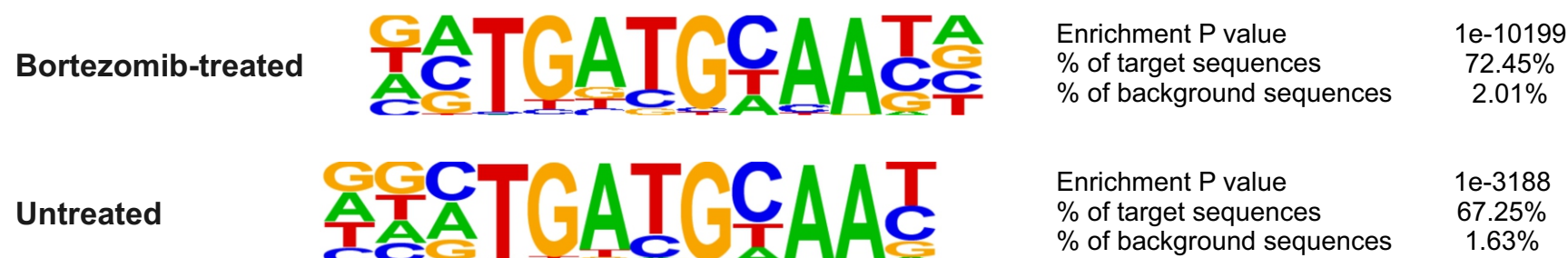

Supplement: S2 Fig — (A) Number of overlapping and unique peaks for the treatments. (B) Fraction of peaks shared between treatments as a function of peak strength. Peaks were ranked by the MACS2 q-value within the library. (C) Motif logos and enrichment statistics for the most significant motif discovered by HOMER de novo motif finding using peak regions called for each treatment. (PDF) [file pgen.1011014.s002.pdf]

Supplementary Figure S4

A

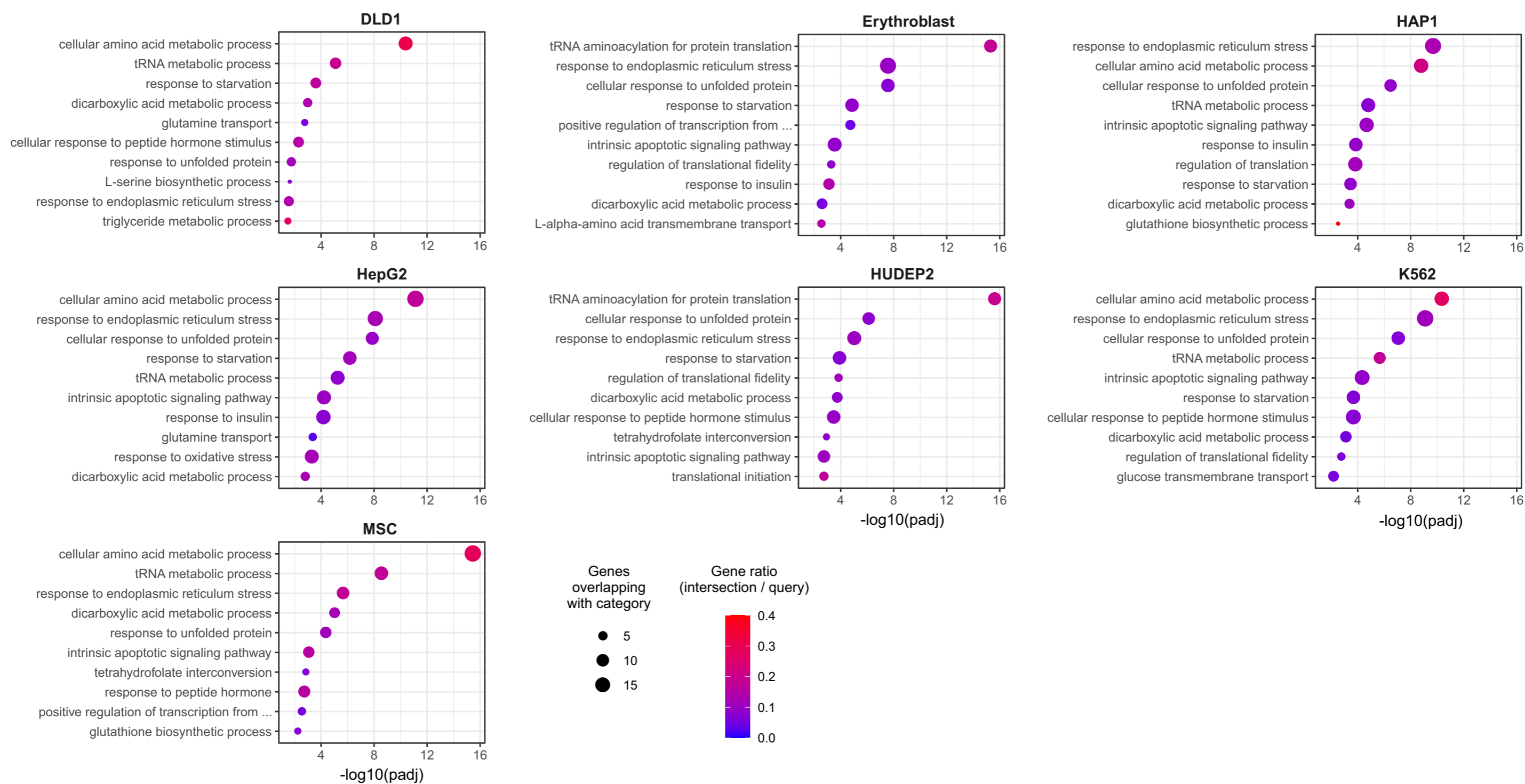

B

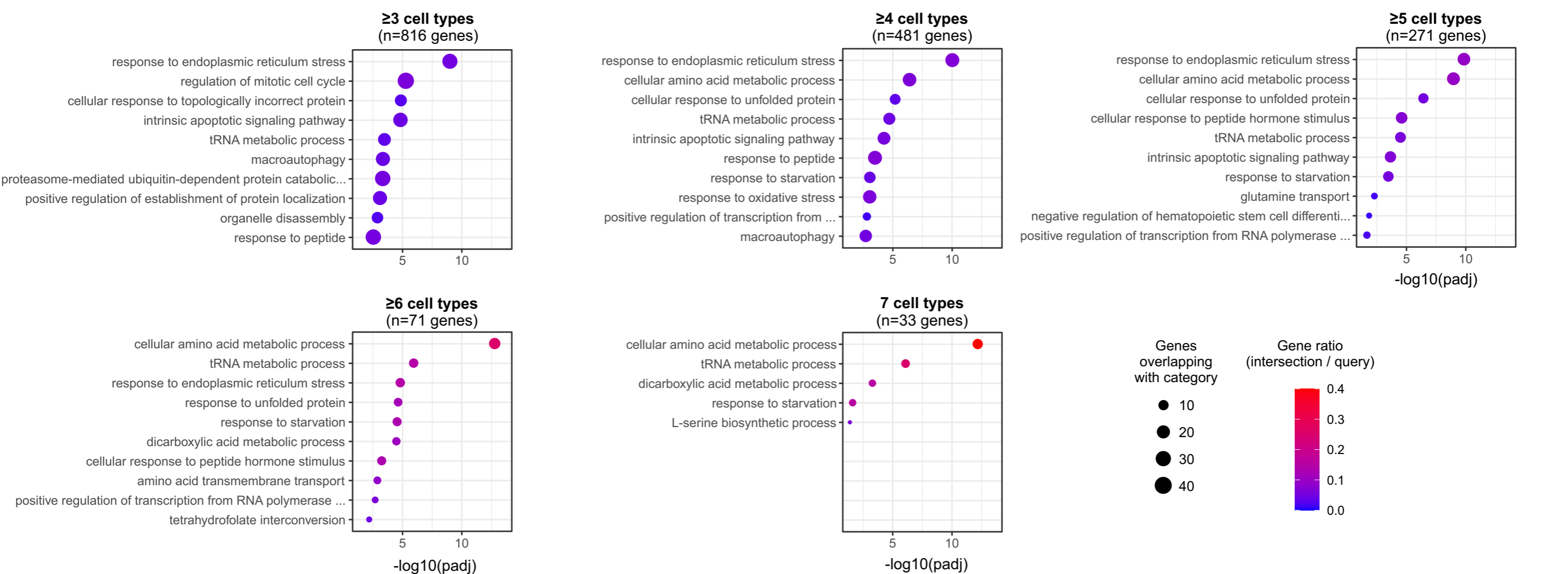

C

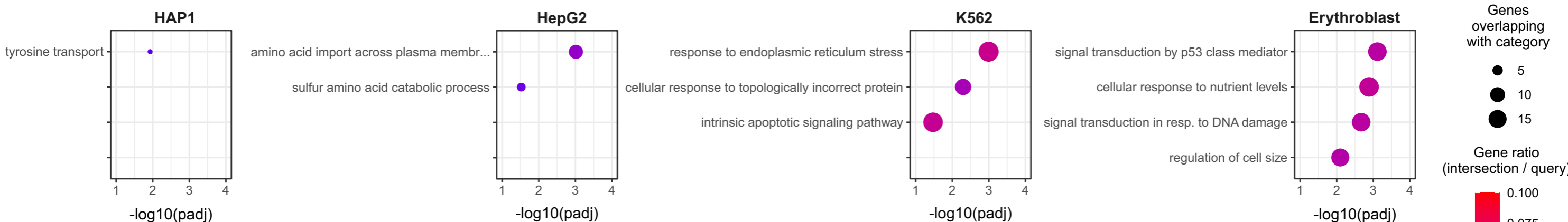

D

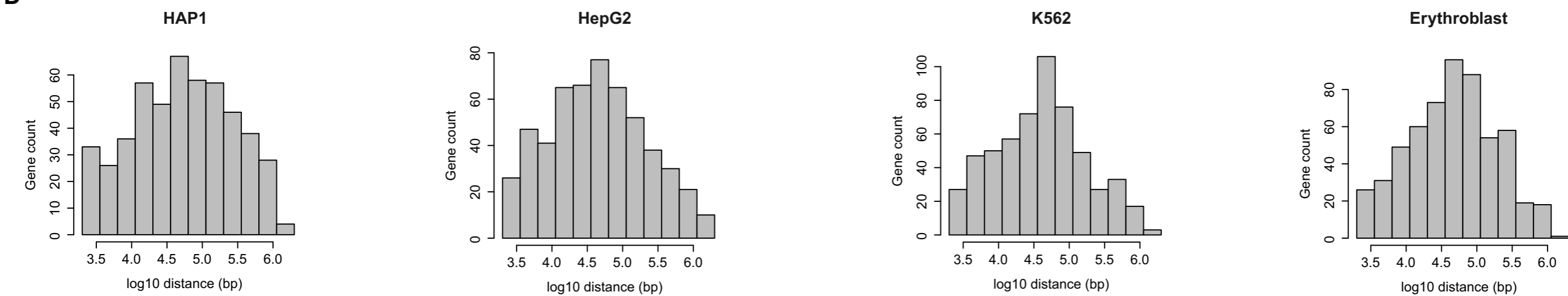

Supplement: S4 Fig — (A) Genes from the 200 strongest TSS/promoter peaks per cell type. (B) Genes with a TSS-proximal ATF4 peak in multiple cell types. (C) Genes connected to the 250 strongest non-promoter peaks (enhancer-gene connections predicted by the ABC model). All four cell types with available ABC model data are shown. ABC connections with score > = 0.015 were retained, and up to top 3 genes by ABC score were considered for each peak. (D) Distances of the regulatory element-gene connections used in panel C. In all panels, one ChIP-Seq library was used to represent each cell type (the library with the most peaks called). (PDF) [file pgen.1011014.s004.pdf]

# Supplementary Figure S5

A

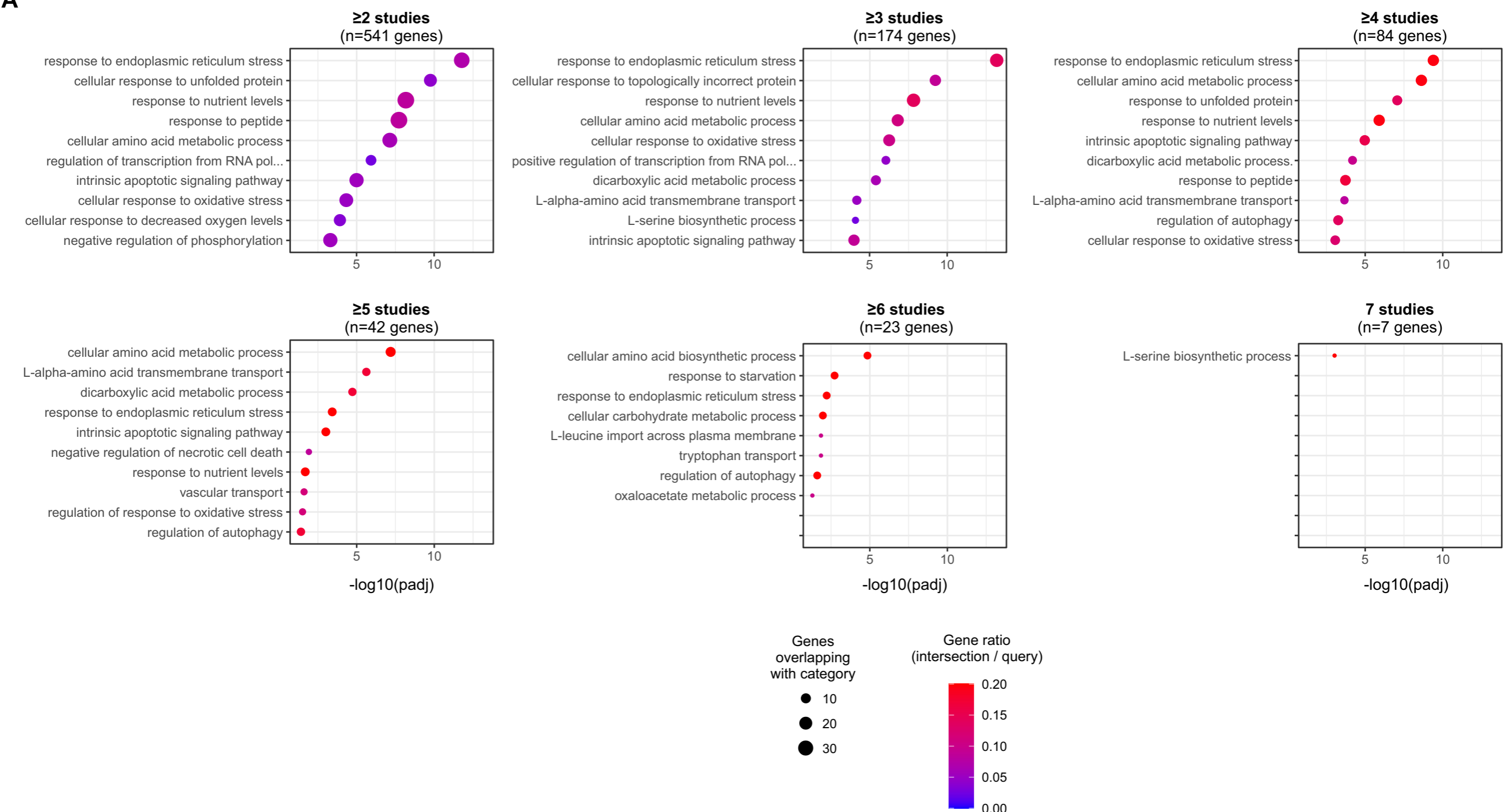

B

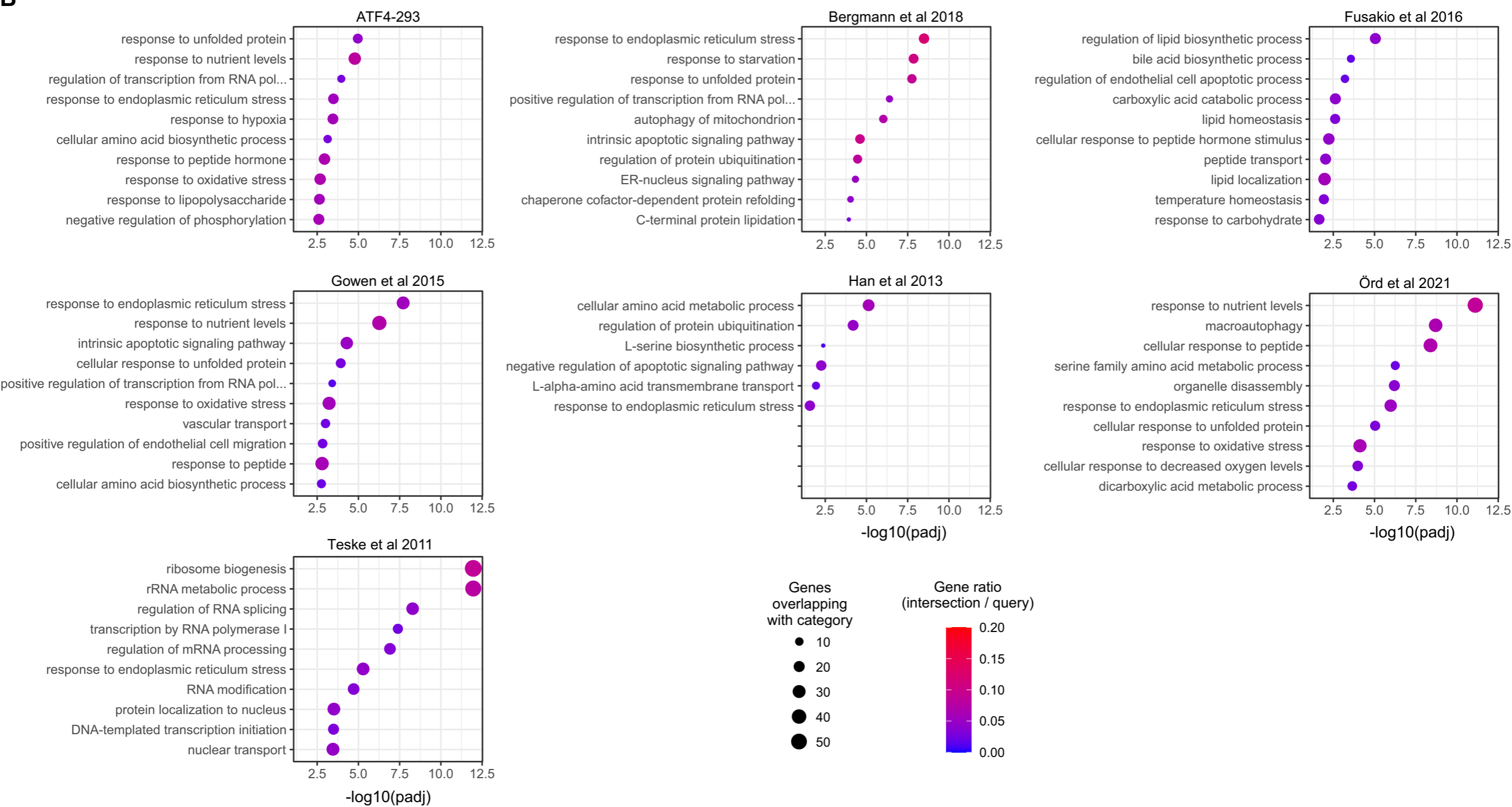

Supplement: S5 Fig — (A) Genes detected in multiple studies. (B) Genes detected in individual studies. (PDF) [file pgen.1011014.s005.pdf]

# Supplementary Figure S7

**A**

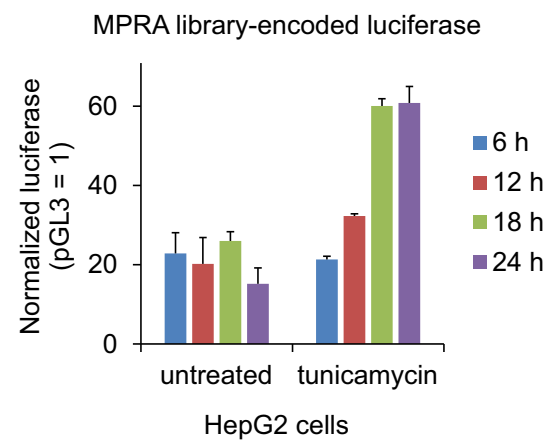

**B**

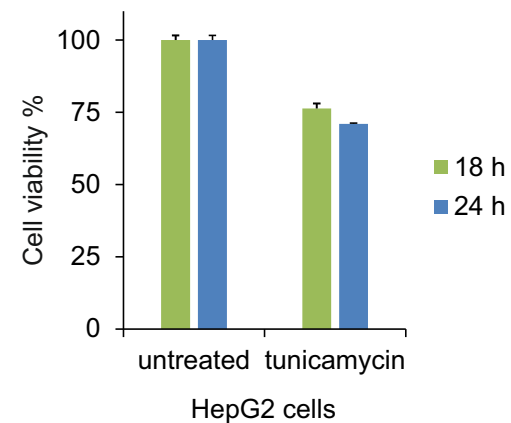

**C**

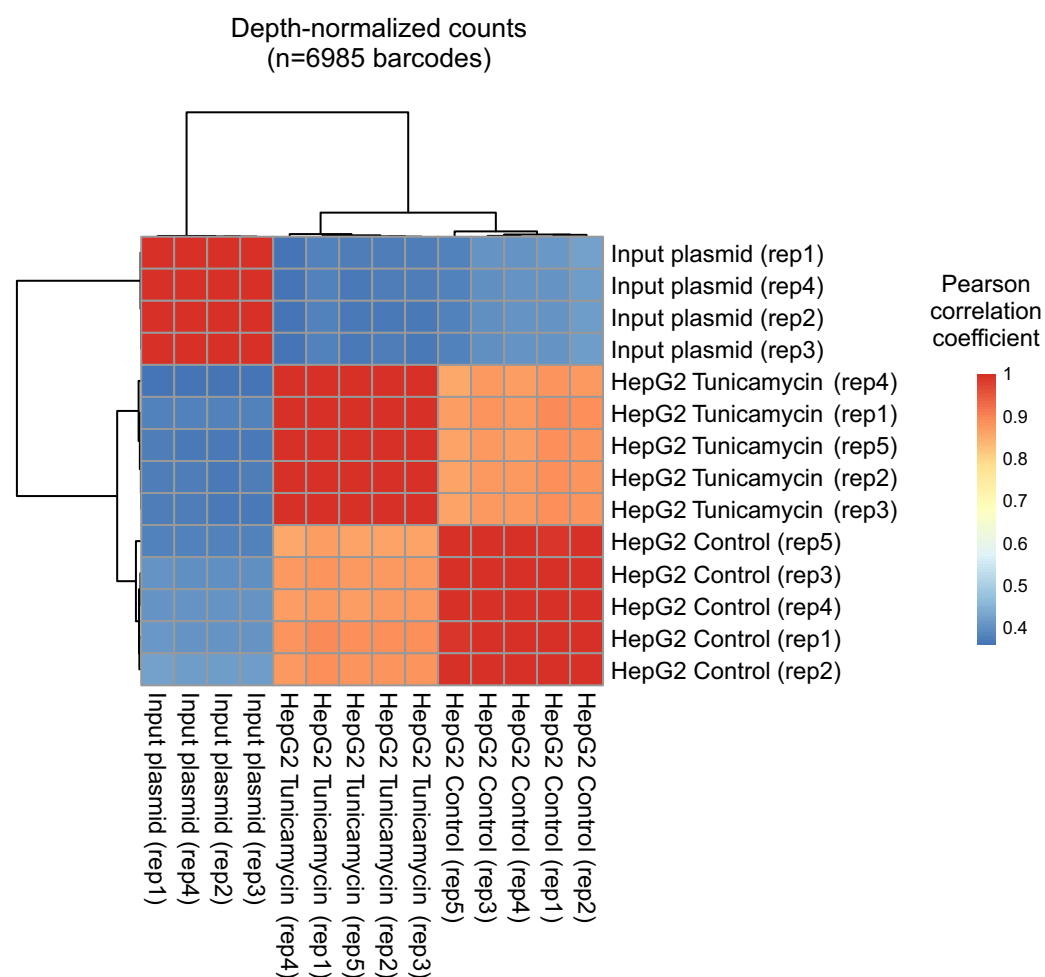

**D**

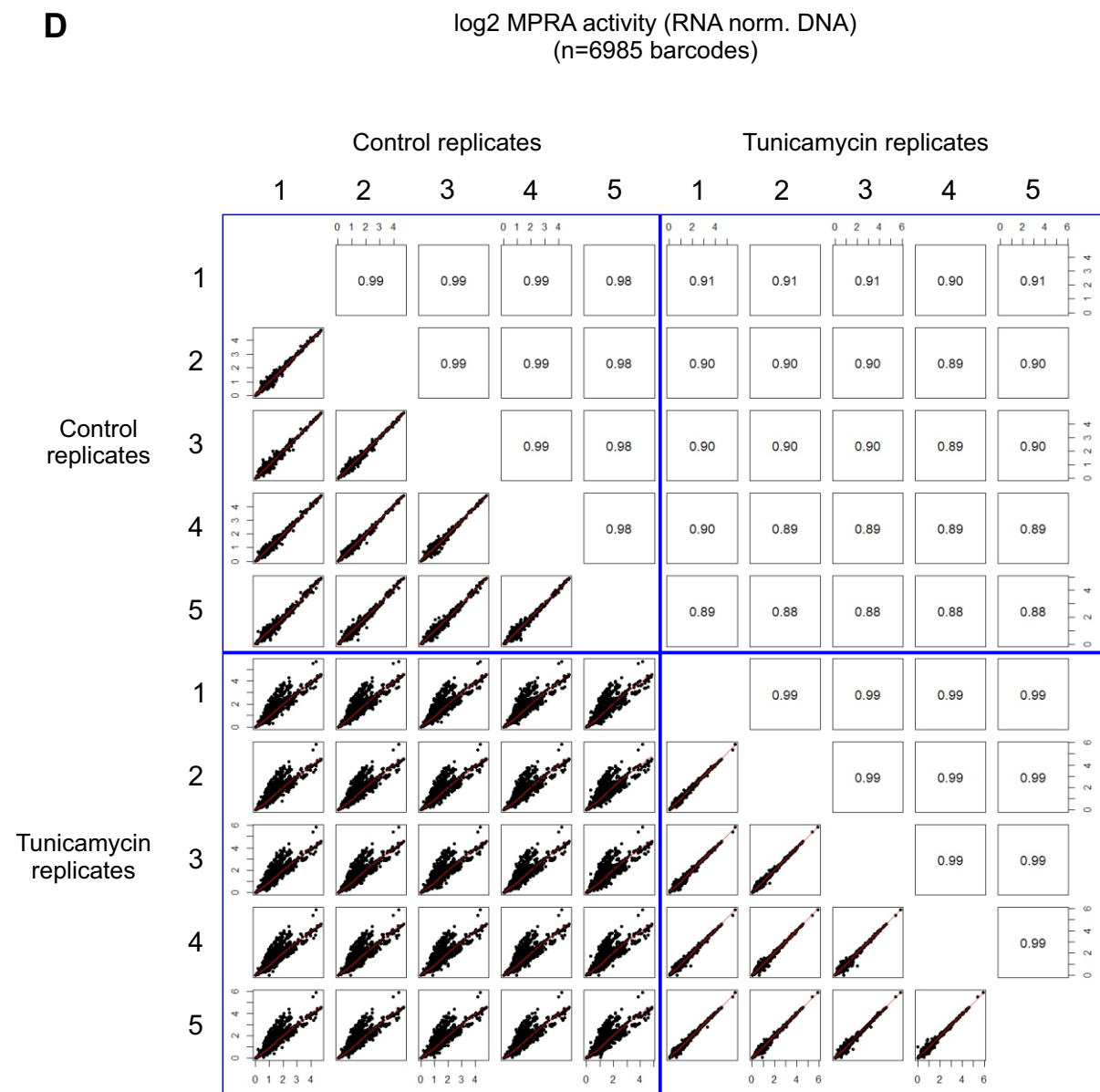

Supplement: S7 Fig — (A) Time-course of luciferase reporter activity of MPRA library (in bulk) in untreated or tunicamycin-treated HepG2 cells. The mean ± SD is shown from 2 experiments performed on separate days. (B) Analysis of cell viability by Alamar Blue assay in HepG2 cells untreated or treated with tunicamycin for 18 or 24 h. The mean ± SD is shown from 2 (untreated) or 3 (tunicamycin) independent experiments. (C) Sample correlation for MPRA barcode counts (normalized for sequencing depth). All 6985 barcodes that passed minimum count filter were used. (D) Sample correlation at the level of log2-transformed ‘MPRA activity’ (RNA signal normalized to input DNA signal). Pairwise Pearson correlation is shown above the diagonal. (PDF) [file pgen.1011014.s007.pdf]

# Supplementary Figure S8

**A**

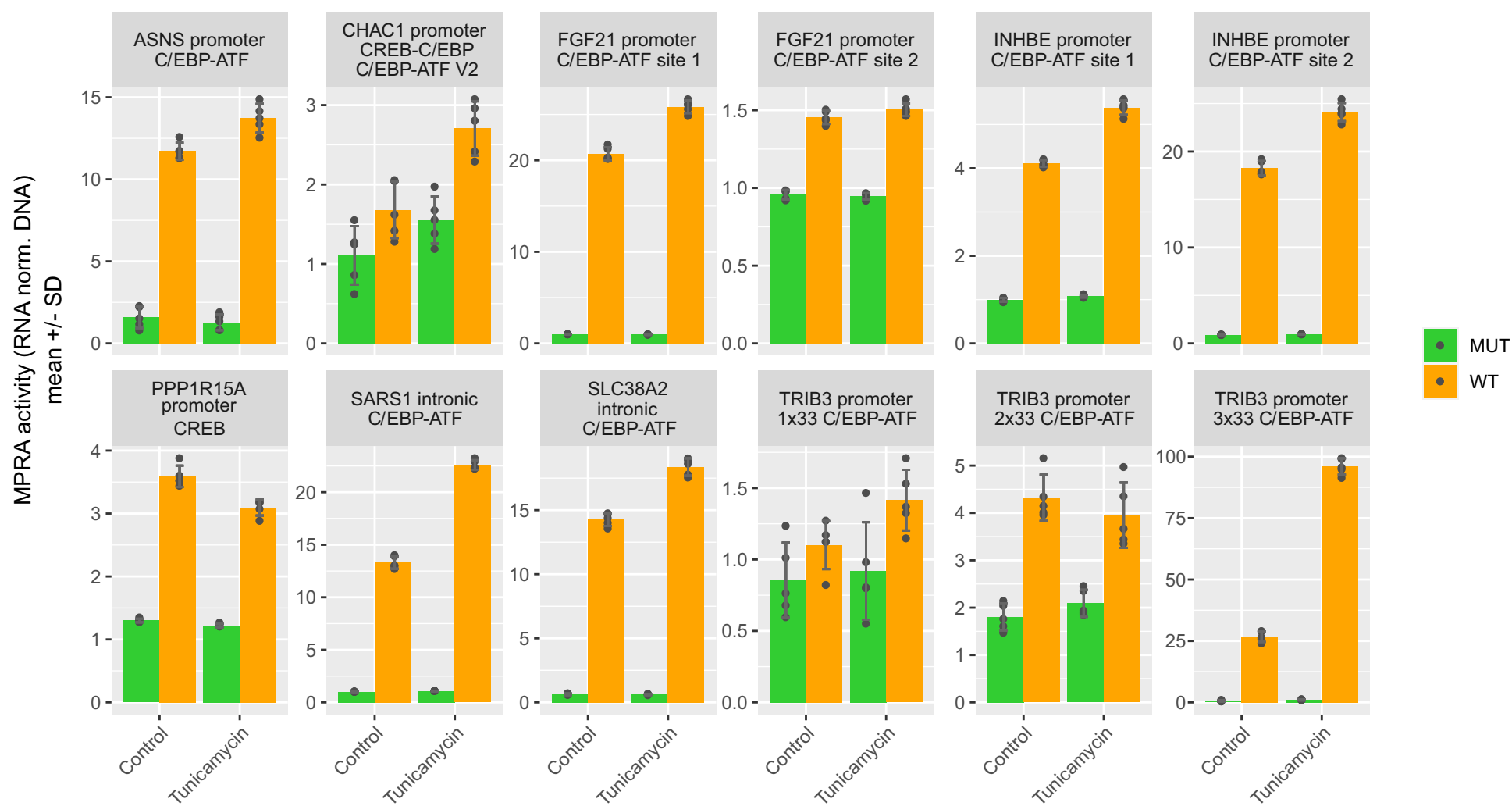

**B**

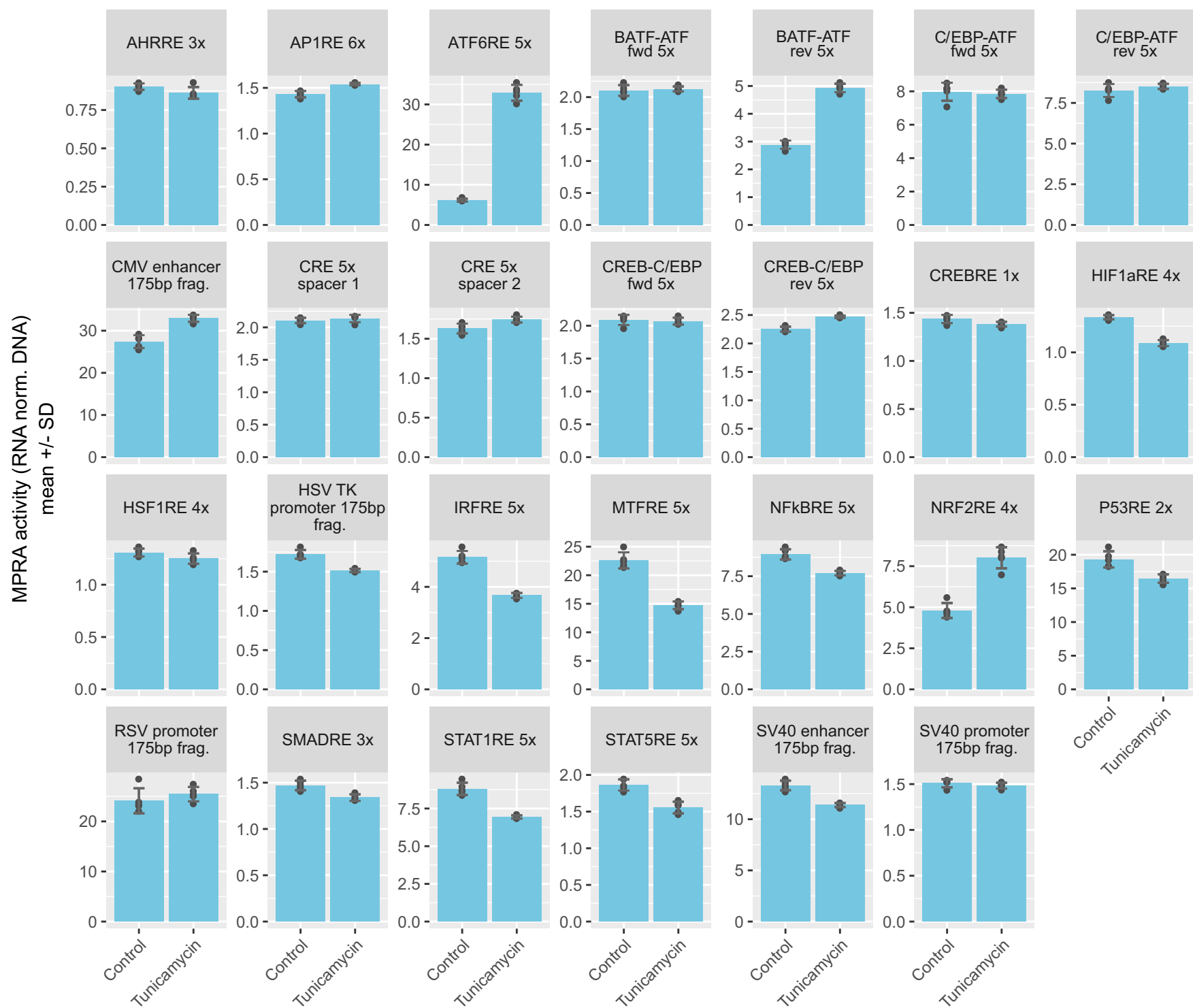

Supplement: S8 Fig — (A) Reported ATF4-responsive elements from human genes. For each element, the wild type (WT) sequence and an engineered mutation (MUT) was assayed. (B) Fragments of viral promoters or enhancers, or artificial sequences containing known transcription factor response elements. In both A and B, the bars show the mean reporter activity (RNA normalized to DNA), the error bars the SD, and the dark gray dots show results from individual experiments (n = 5 per condition). (PDF) [file pgen.1011014.s008.pdf]

Supplementary Figure S9

**A**

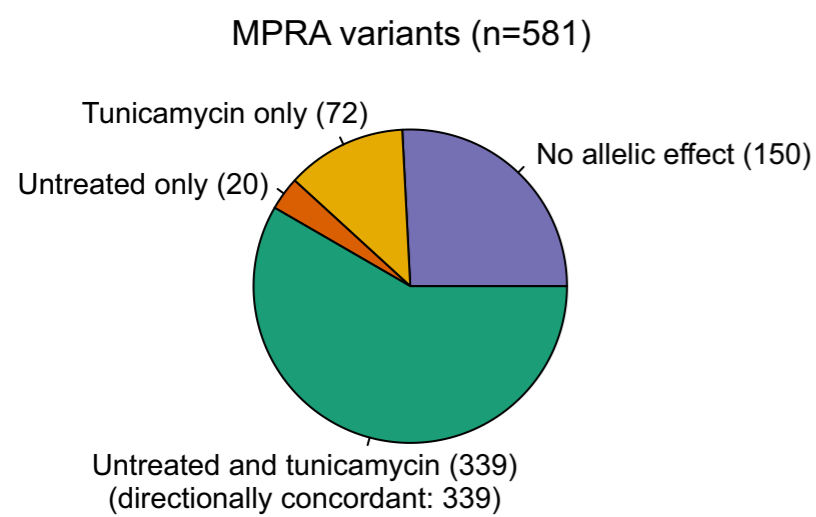

**B**

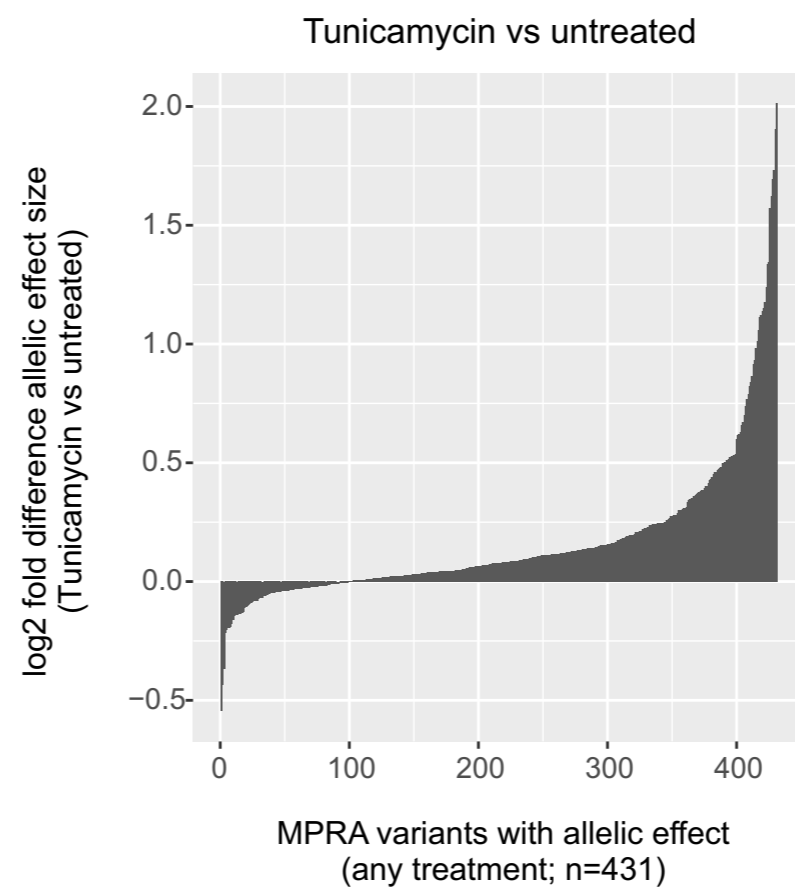

**C**

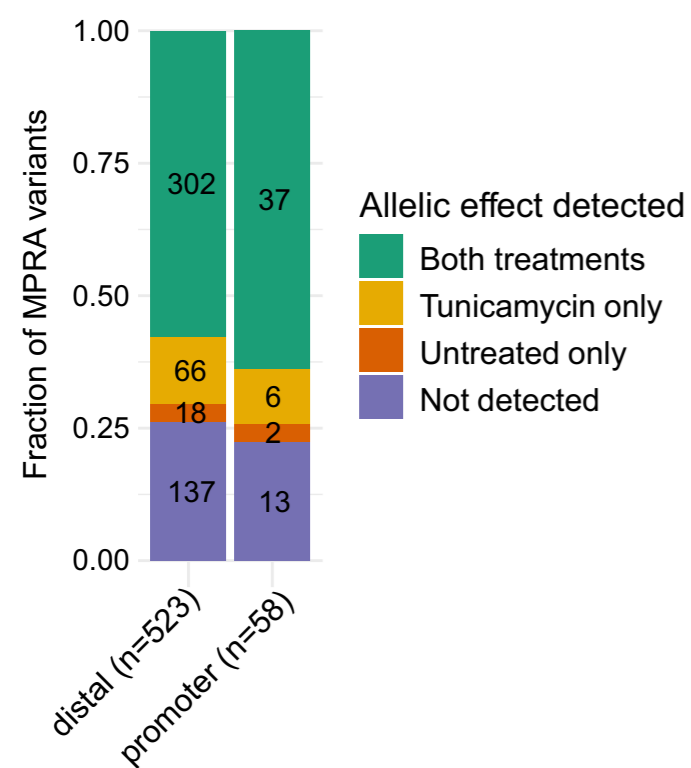

**D**

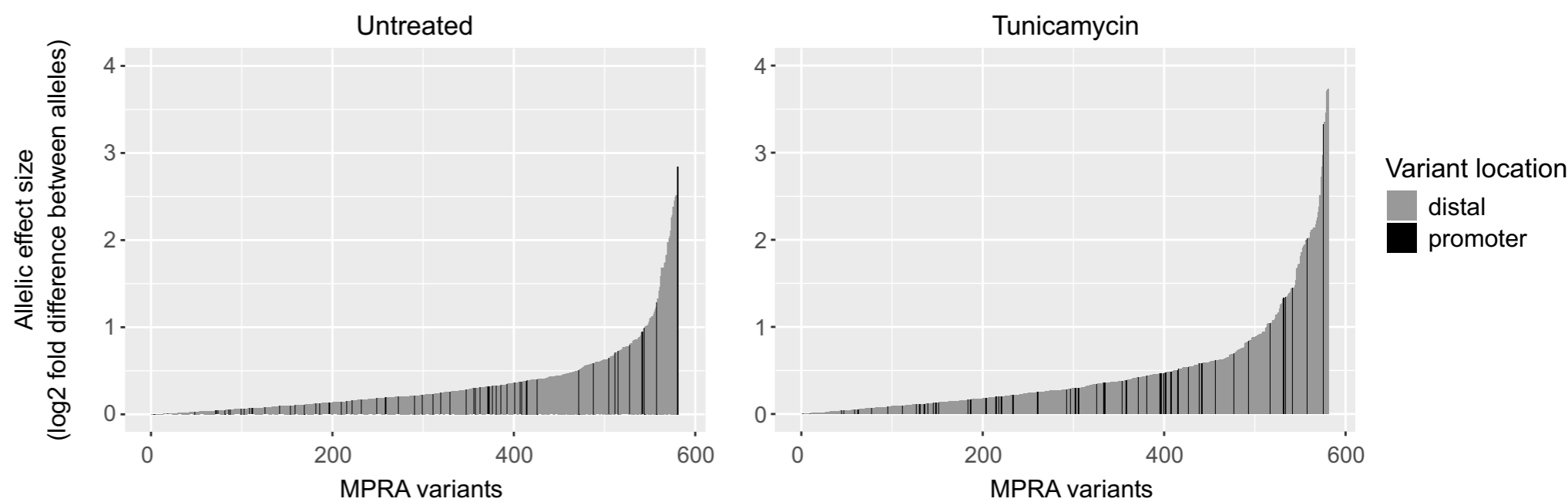

**E**

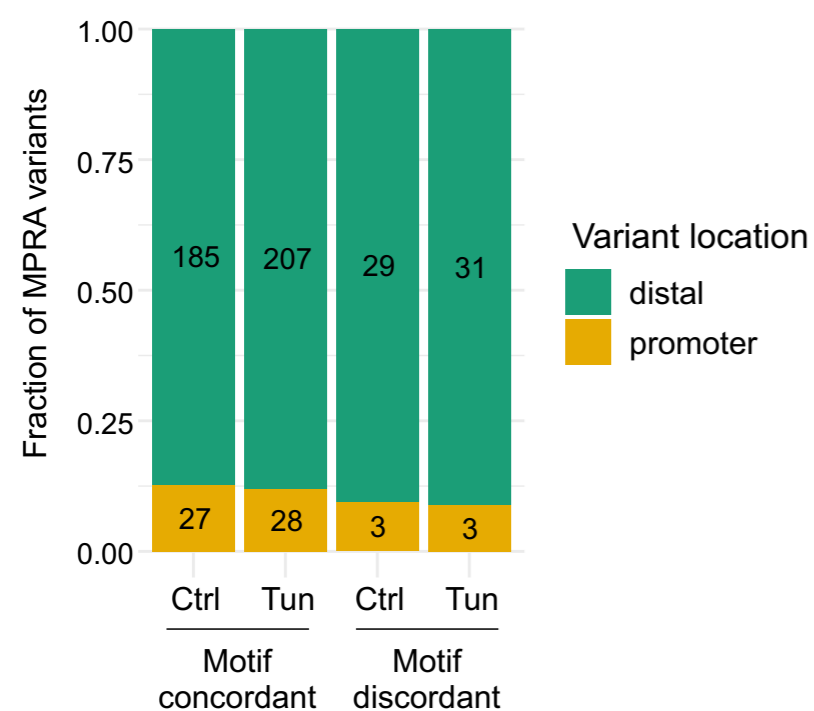

**F**

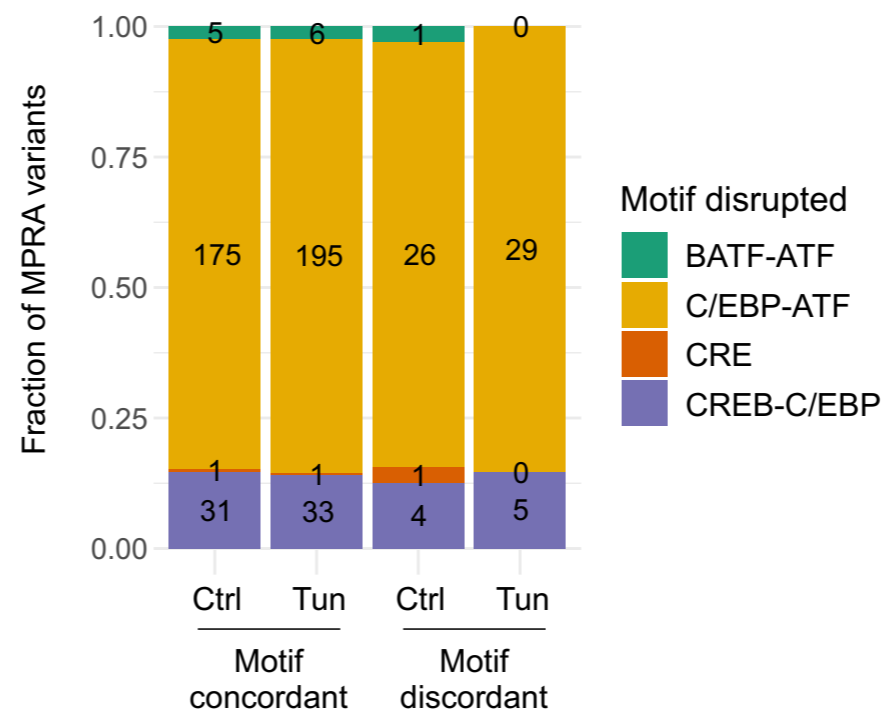

Supplement: S9 Fig — (A) Number of MPRA variants with a significant (MPRAnalyze FDR < 0.05) allelic effect by treatment. (B) Effect of treatment on allelic effect size. All MPRA variants with a significant allelic effect in at least one treatment were included and are shown ranked by treatment difference. (C) Allelic effect detection by genomic location (promoter or distal). (D) Distribution of individual allelic effect sizes, showing the regulatory element type. All 581 variants were included and shown ranked by effect size. (E and F) MPRA allelic effects by directional concordance with motif disruptions predicted from sequence. Significant MPRA allelic effects were categorized as motif-concordant if the higher activity allele created a known ATF4 binding motif, and motif-discordant if the higher activity allele disrupted an ATF4 motif. Motif concordance is shown stratified by genomic location (E) and type of motif disrupted (F). In panels C, E and F, the number of variants in forming each set is reported on the bar segments. Promoter regions were defined as TSS +/- 1kb. Ctrl, Control (untreated); Tun, Tunicamycin. (PDF) [file pgen.1011014.s009.pdf]

# Supplementary Figure S10

**A**

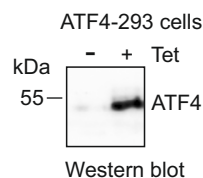

**B**

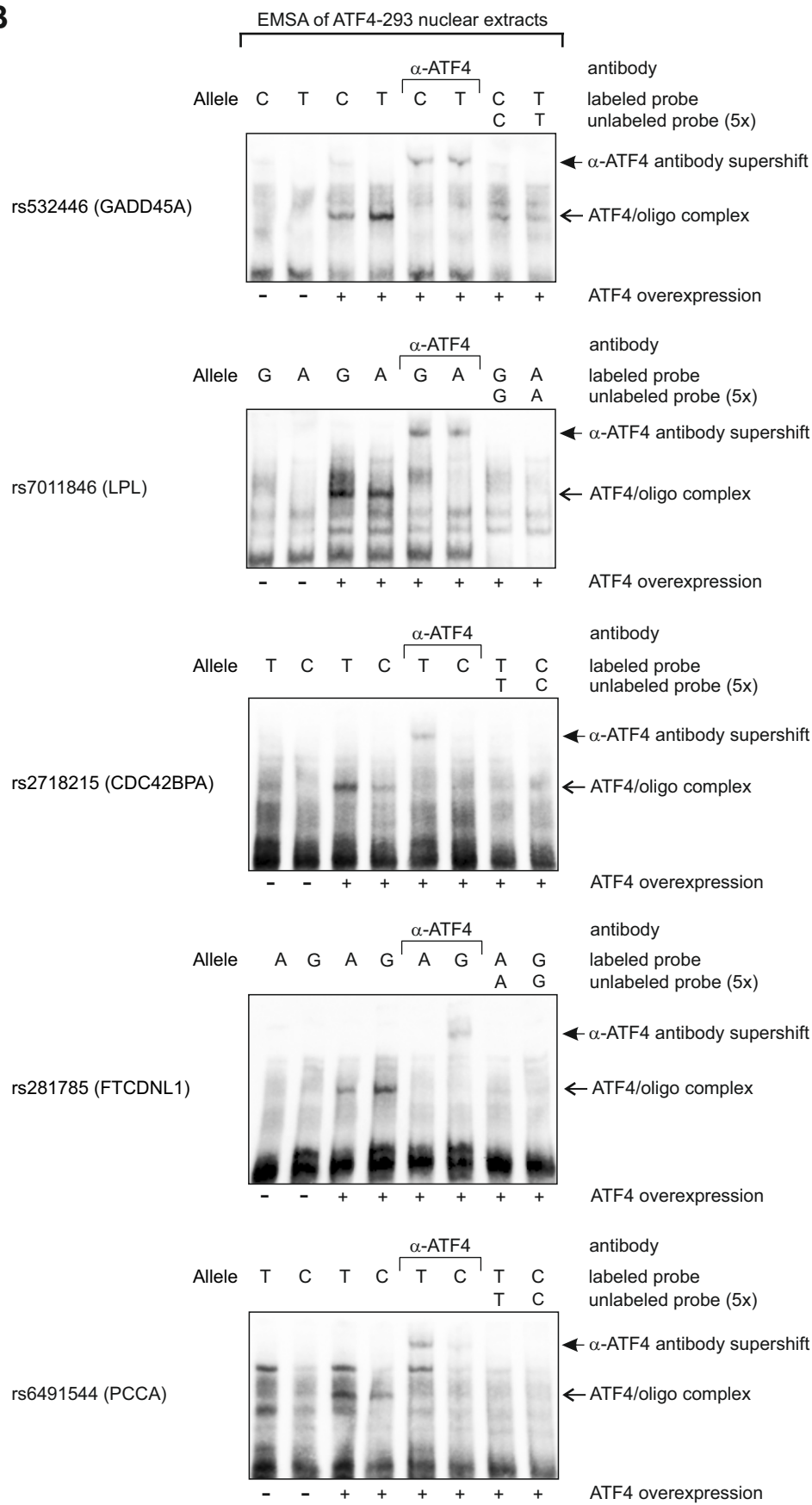

Supplement: S10 Fig — Cells were untreated (-) or treated with tetracycline (+) to induce ATF4 overexpression from a stably integrated tetracycline-upregulated expression construct. (A) ATF4 protein level in nuclear extracts (20 μg per line) determined using Western blotting. (B) EMSA of ATF4 DNA binding ability. Biotin-labeled oligonucleotides corresponding to either the ref or the alt allele of the SNP were used as probes. In lanes labeled α-ATF4, antibody targeting ATF4 was included in the reaction. Where indicated, unlabeled competitor probe was added to the reaction in 5-fold excess. (PDF) [file pgen.1011014.s010.pdf]
