# Supplementary material for: Genome-wide census of ATF4 binding sites and functional profiling of trait-associated genetic variants overlapping ATF4 binding motifs
Source: PLoS Genet. 2023 Oct 31;19(10):e1011014. doi: 10.1371/journal.pgen.1011014 (PMC10637723; doi:10.1371/journal.pgen.1011014)

Supplementary Figure S3

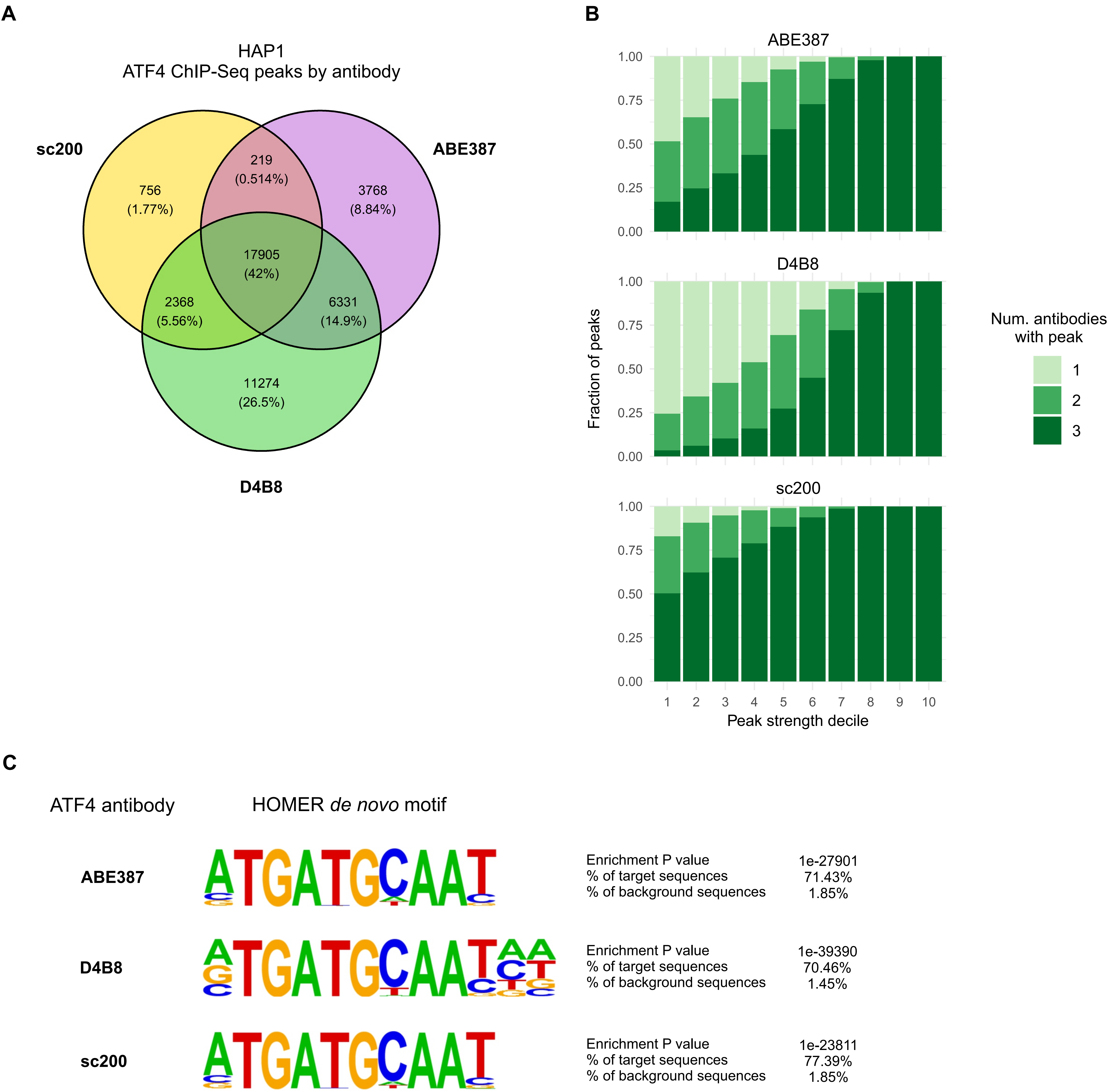

Supplement: S3 Fig — (A) Number of overlapping and unique peaks for the different antibodies. (B) Fraction of peaks shared between antibodies as a function of peak strength. Peaks were ranked by the MACS2 q-value within the library. (C) Motif logos and enrichment statistics for the most significant motif discovered by HOMER de novo motif finding using the peak regions called for each antibody. (PDF) [file pgen.1011014.s003.pdf]
