# Supplementary material for: Genome-wide census of ATF4 binding sites and functional profiling of trait-associated genetic variants overlapping ATF4 binding motifs
Source: PLoS Genet. 2023 Oct 31;19(10):e1011014. doi: 10.1371/journal.pgen.1011014 (PMC10637723; doi:10.1371/journal.pgen.1011014)

Supplementary Figure S6

A

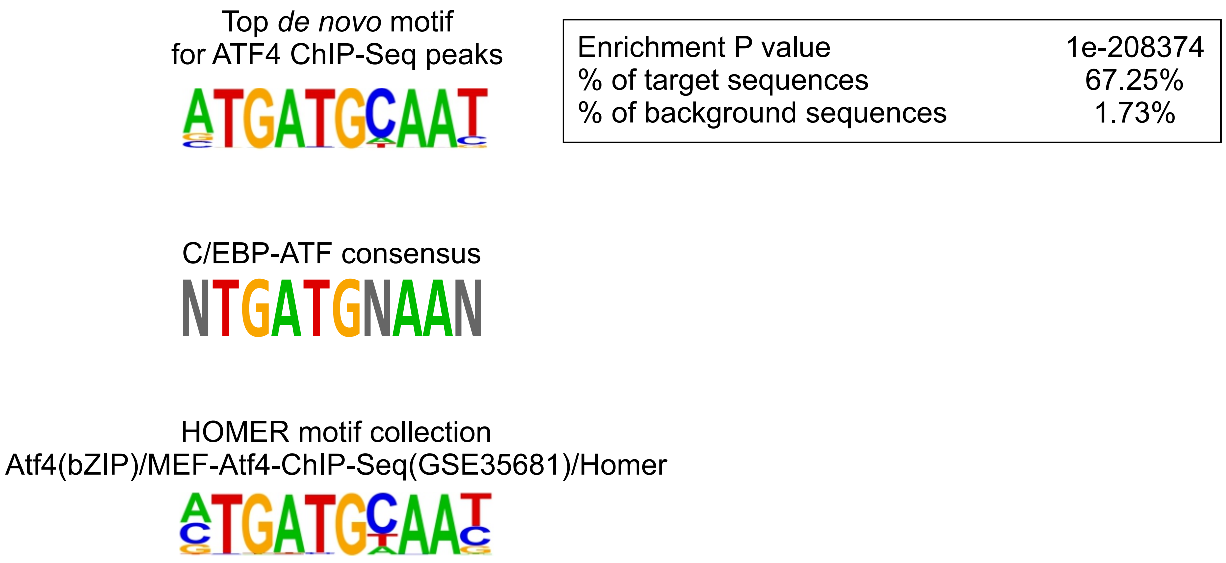

B

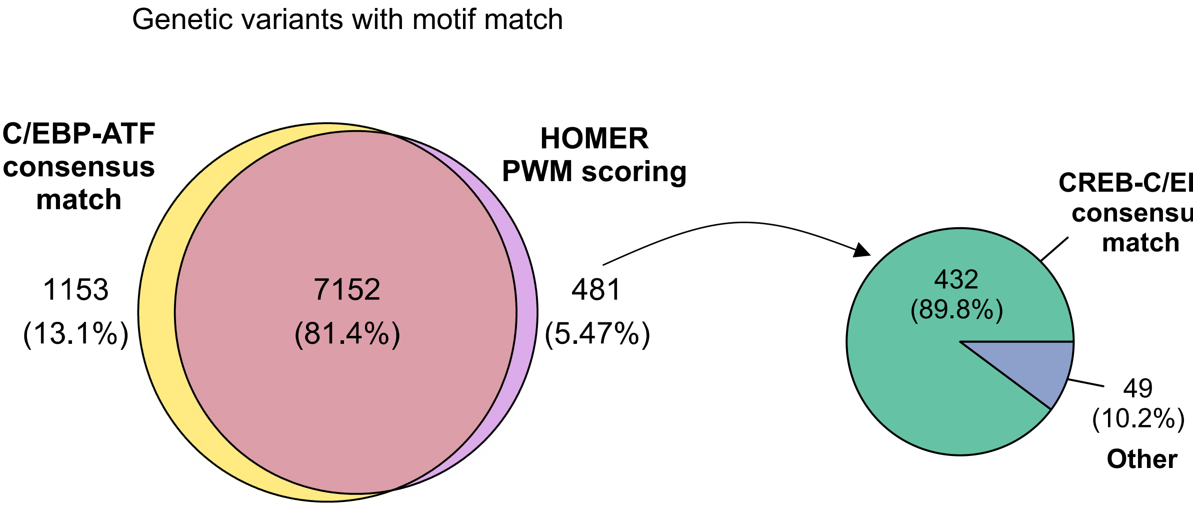

C

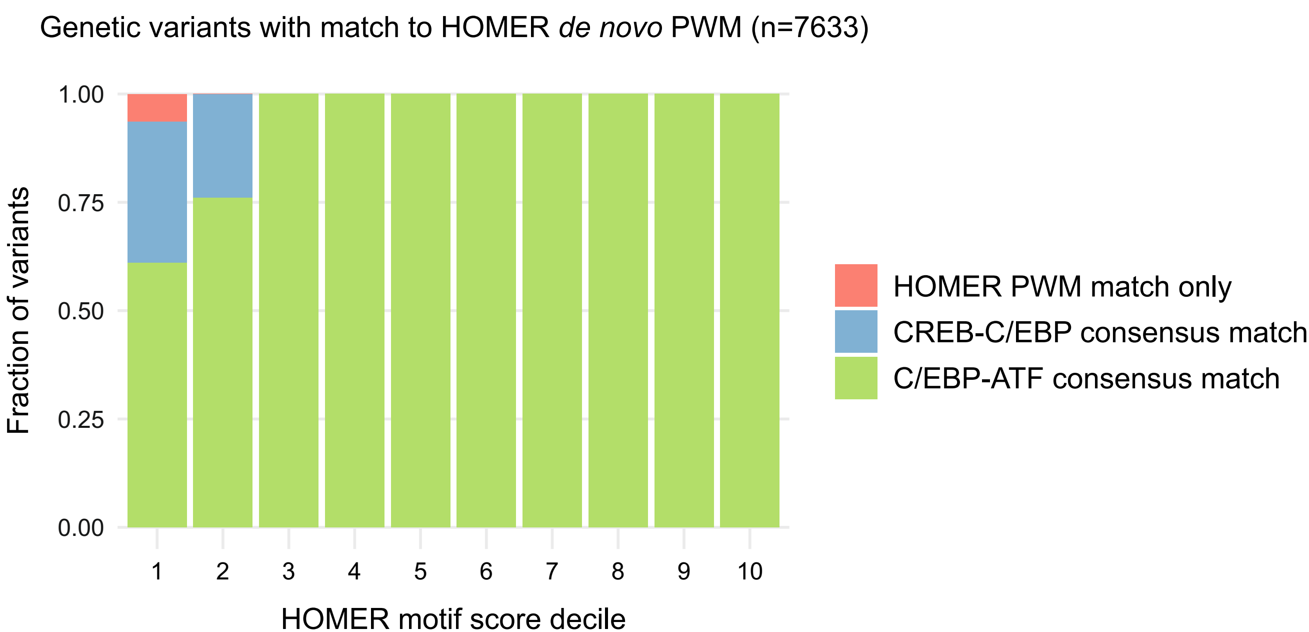

Supplement: S6 Fig — (A) Motif logo and occurrence statistics of the most strongly enriched de novo motif identified by HOMER within ATF4 peaks in the current study. The C/EBP-ATF consensus sequence (NTGATGNAAN) and the ATF4 motif from the HOMER motif catalog are shown for comparison. (B) Number of genetic variants found as motif overlapping. All genetic variants falling within ATF4 ChIP-Seq peaks (merged across all ATF4 IP libraries) were considered with all variant alleles when searching for motif matches. The CREB-C/EBP consensus was defined as NTGACGNAAN. (C) HOMER motif match score distribution for all variants that matched the HOMER de novo PWM for ATF4 with any allele. The highest scoring allele for each variant was retained. The fraction of variants that matched a consensus sequence is shown. (PDF) [file pgen.1011014.s006.pdf]
